# Circular RNA circFat3 as a biomarker for construction of postmortem interval Estimation models in mouse brain tissues at multiple temperatures

**DOI:** 10.1038/s41598-025-07998-0

**Published:** 2025-07-01

**Authors:** Binghui Song, Jiewen Fu, Jingliang Cheng, Sawitree Chiampanichayakul, Songyot Anuchapreeda, Junjiang Fu

**Affiliations:** 1 Department of Medical Technology, Faculty of Associated Medical Sciences, Chiang Mai University, Chiang Mai, 50200 Thailand; 2 Key Laboratory of Epigenetics and Oncology, The Research Center for Preclinical Medicine, Southwest Medical University, Luzhou, 646000 Sichuan China; 3 Laboratory of Precision Medicine and DNA Forensic Medicine, The Research Center for Preclinical Medicine, Southwest Medical University, Luzhou, 646000 Sichuan China; 4 Center of Excellence in Pharmaceutical Nanotechnology, Chiang Mai University, Chiang Mai, 50200 Thailand; 5 Laboratory of Forensic DNA, the Judicial Authentication Center, Southwest Medical University, Luzhou, 646000 Sichuan China

**Keywords:** Postmortem interval (PMI), Circular RNA (circRNA), Forensic medicine, PMI Estimation model, Genetic markers, Non-coding RNAs

## Abstract

Circular RNAs (circRNAs) are conserved, abundant, stable, and specifically expressed in mammals. The postmortem interval (PMI) estimation is crucial in forensic medicine, particularly for case investigation and civil action. CircRNAs may serve as ideal PMI biomarkers. However, no research has explored PMI estimation in the brain using circRNAs. The total RNA, including circRNA, was sampled from mouse brain tissues at multiple temperatures (4℃, 25℃, and 35℃). The semi-quantitative reverse transcription (RT)-PCR and real-time quantitative PCR (RT-qPCR) were used to test the postmortem degradation levels at different PMIs. As a result, we found circFat3 is highly and specifically expressed in mouse brain tissue, with postmortem levels significantly correlated with PMI across multiple temperatures. In addition, mt-co1 and 28 S rRNA demonstrated stability under various temperature conditions, supporting their use as reliable reference genes for PMI models. Moreover, the error rates showed that the circFat3/28S rRNA model was more accurate at 4℃. The circFat3/mt-co1 and circFat3/28S rRNA models provided slightly better predictions for short-term and long-term PMI, respectively at 25℃, while the circFat3/mt-co1 model was more accurate at 35℃. The combined application of the two reference genes was beneficial primarily for long-term PMI estimation. Furthermore, the validation results confirmed that these models were more accurate for long-term PMI estimation. Thus, our mathematical models were constructed at multiple temperatures based on circFat3 and these two reference genes. Taken together, this is the first study to identify circRNA circFat3 as a novel biomarker that may serve as a complementary tool for PMI estimation.

**Supplementary Information:**

The online version contains supplementary material available at 10.1038/s41598-025-07998-0.

## Introduction

Postmortem interval (PMI) refers to the period between the discovery or investigation of a cadaver and the actual time of death. PMI estimation remains a key challenge in forensic identification, and it currently relies on morphological techniques. However, these methods are susceptible to subjective influences and environmental factors^1,2^. With the continuous development of technologies related to biochemistry and molecular biology, molecular markers including DNA^3^, RNA^4^, and proteins^5^ have become new tools for PMI estimation. Over the years, various RNAs such as messenger RNA (mRNA), ribosomal RNA (rRNA), and microRNA (miRNA) have been tested for PMI estimation. The results of related studies have demonstrated their potential utility in PMI estimation for forensic cases^6,7^. In recent years, the rise of RNA-seq technology and the development of computational pipelines have accelerated research on circular RNA (circRNA)^8–10^. Owing to its structural stability, tissue specificity, and developmental-stage specificity, circRNA holds promising potential for application in PMI estimation, yet related studies are very scarce.

As early as 1976, Sanger et al. first discovered circRNA in viroids, but it did not receive much attention^11^. For many years it was regarded as a product of splicing errors, and it was only with the maturation of sequencing technology that circRNAs were found to play an important role in organisms^12^. It was not until the early 2010s that circRNA research gained significant attention. CircRNA is an endogenous biomolecule in which pre-mRNA undergoes back-splicing to form a circular structure, and it is primarily distributed in the exosome and cytoplasm^13,14^. Due to its closed circular structure, it is less susceptible to the influence of ribonuclease R (RNase R) compared to other linear RNAs^15^. Research has shown that the half-life of most exon circRNAs is more than 48 h in cells, while the average half-life of mRNA is 10 h^16^. The length of human circRNAs is usually less than 1500 nucleotides, with an average length of around 500 nucleotides^17^. Functionally, circRNAs act as miRNA sponges, primarily through the competitive endogenous RNA (ceRNA) mechanism^18^. CircRNAs may act as ceRNAs to inhibit the function of miRNAs by directly or indirectly binding to them, thereby relieving the inhibitory effect of miRNAs on target genes and promoting the expression of these target genes. In addition, circRNAs play important roles in regulating transcription, encoding proteins, participating in translation, regulating cell cycle, and inducing apoptosis. They have been widely studied in the context of physiological processes, cancers, immune regulation, cardiovascular diseases, and metabolic disorders^19–21^.

At present, the research on circRNA has been conducted in forensic medicine, including age estimation, identification of biological sample sources, estimation of bloodstain age, analysis of the cause of death, and the identification of monozygotic twins. Due to the widespread presence of ribonucleases, it is generally believed that RNA is more susceptible to degradation than DNA in vitro and after death. However, studies have shown that circRNAs are highly stable. Although circRNA shares the same primary structure as linear RNA, it is not easily degraded by RNase R^22^. Compared with general nucleic acid molecules, circRNA has a closed-loop structure with stable expression and a long half-life, making it more suitable for forensic research. Its high stability and tissue-specificity make circRNA potentially valuable for PMI estimation^23^. It has now been demonstrated the human brain exhibits relatively high expression and specificity of circRNAs and that circRNAs are abundantly expressed in mammalian brains, including the human brain, compared to other tissues^23–27^. CircRNAs are highly enriched in synapses, and some conserved circRNAs are expressed in both mice and humans. In addition, the relatively closed anatomical location of brain tissue within the skull makes it less affected by external factors compared to other tissues that are susceptible to exposure to various environments such as skin. Brain tissue may serve as an ideal tissue for PMI estimation due to its enriched and conserved circRNA and low exposure to external factors. However, there are no studies on circRNA in PMI in brain tissues. Therefore, the changes in postmortem levels of circRNA in brain tissue at different PMIs, as well as the potential application of circRNA in forensic PMI estimation, require further exploration.

This study aims to explore the potential of circRNAs for forensic PMI estimation to identify effective circRNA markers. Beginning with brain tissue, human-mouse homologous circRNAs were screened from existing databases, and specific primers were designed. By constructing mouse models with different PMIs under various environment conditions, including low temperature (4℃), room temperature (25℃), and high temperature (35℃), the expression level of circRNAs was detected using experiments such as semi-quantitative reverse transcription-polymerase chain reaction (RT-PCR) and real-time quantitative PCR (RT-qPCR). The mathematical models for PMI estimation were then established, and the accuracy was verified using mouse samples with known times of death. The successful development of these mathematical models based on circFat3 can provide a practical and effective tool for PMI estimation, which is of great significance for the application of circRNAs in forensic science and technology.

## Results

### The selection of human-mouse homologous circRNA

**Fig. 1:**
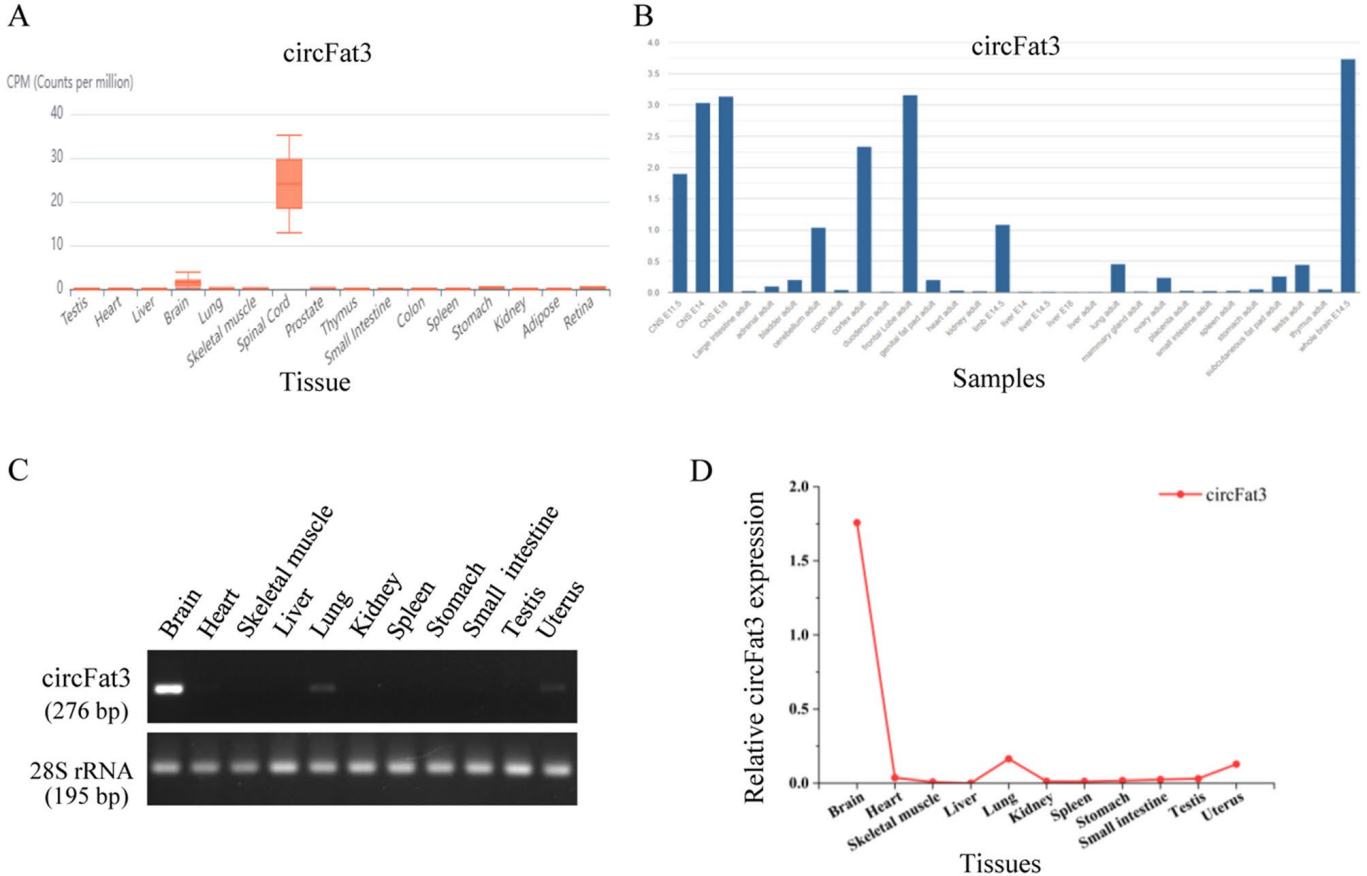
The expression of circFat3 in multiple mouse tissues. (**A**) The expression of circFat3 (mmu_circ_0001746) in multiple tissues based on the circAtlas. (**B**) The expression of circFat3’s host gene in different tissues based on the NCBI database. (**C**) The electrophoretic results of circFat3 in 11 tissues. (**D**) The relative expression of circFat3 in 11 different tissues.

Relevant literature was used to preliminarily screen for circRNAs that are highly expressed and specific in mouse brain tissue. Candidate circRNAs with highly expressed parental genes were identified in the NCBI database. Based on mouse information, databases such as circBase and circBank were used to verify the existence of homologous human circRNAs, and the GTEx database was used to identify circRNAs with highly expressed parental genes. Based on the sequencing data from these sources, several circRNA molecules with high expression or strong tissue specificity in brain tissue were identified. Notably, circFat3 and its host gene exhibited higher expression levels in mouse brain tissue (Fig. 1A,B). Moreover, the expression of hsa_circ_0000348 and the *FAT3* gene is also high in human brain tissue (Supplementary Fig. S1A,B). PCR amplification and agarose gel electrophoresis were then performed. Based on the agarose gel electrophoresis results, circFat3 exhibited high specificity in mouse brain tissue (Fig. 1C). The relative expression of Fat3 across 11 different tissues was calculated using the semi-quantitative RT-PCR (Fig. 1D). The results indicated that circFat3 was highly expressed and specific in mouse brain tissue. In addition, the sequences of mmu_circ_0001746 and hsa_circ_0000348 were obtained from circBase, with the full length of 3,306 bp and 3,309 bp, respectively. Homology analysis indicated that these circRNAs exhibited strong conservation between humans and mice (Supplementary Fig. S1C). Finally, the human-mouse homologous and tissue-specific circRNA mmu_circ_0001746 (circFat3), along with its human homologous hsa_circ_0000348, were identified by RT-PCR in our study (Data not shown). The protein encoded by this gene is Protocadherin Fat 3.

### The validation of circFat3

**Fig. 2:**
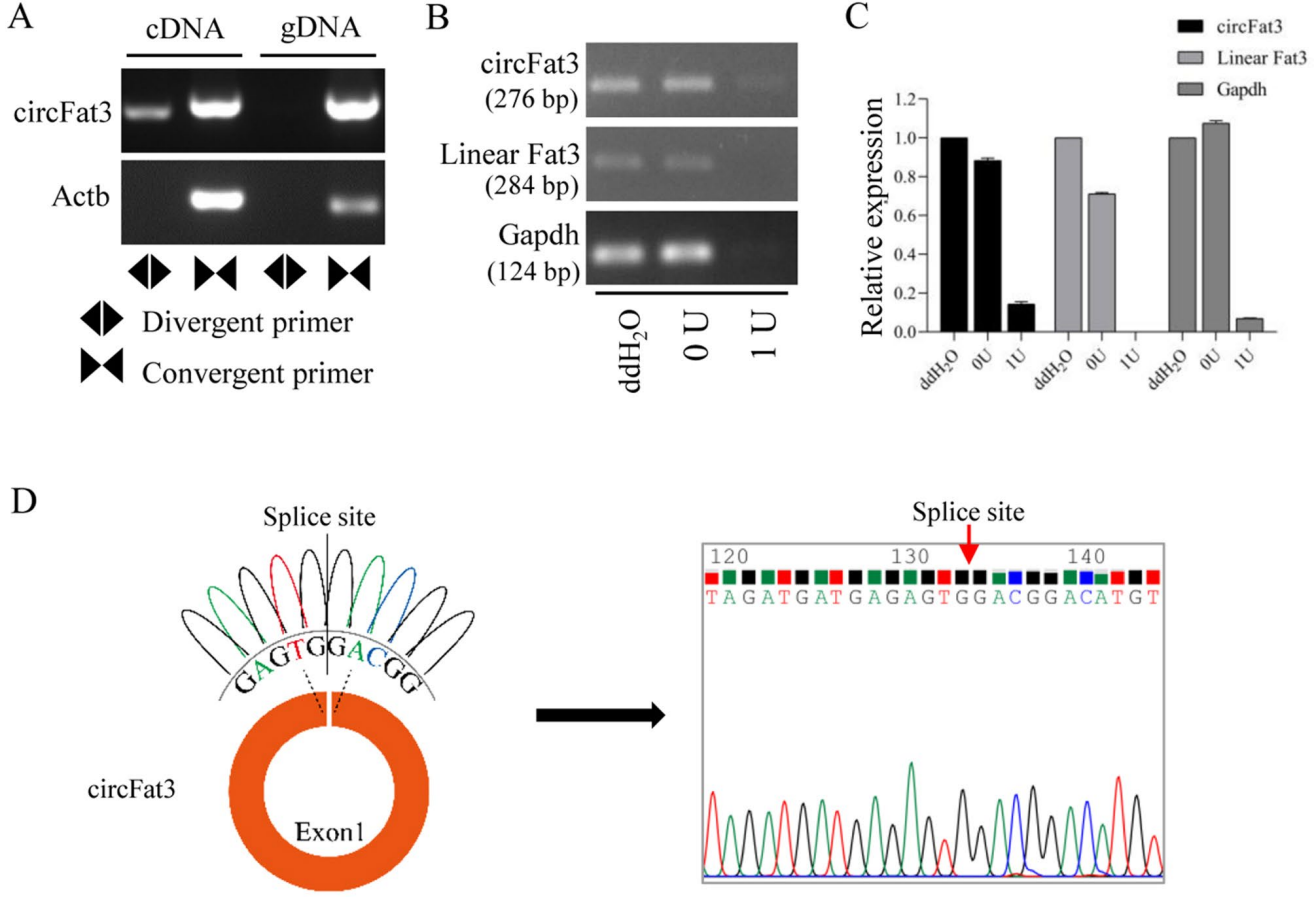
Validation of circFat3 in mouse brain tissue. (**A**) The analysis using convergent and divergent primers of circFat3 in cDNA and gDNA samples. For circFat3, the product lengths of divergent and convergent primers were 276 bp and 284 bp, respectively. (**B**) The RNase R digestion analysis of circFat3, linear Fat3, and *Gapdh* in ddH_2_O group, 0 U group and 1 U group. (**C**) The data analysis of the relative expression of circFat3, linear Fat3, and *Gapdh* after the RNase R digestion. (**D**) The structure of circFat3 and the results of Sanger sequencing.

Divergent and convergent primers were used to assess the expression of circFat3 and *Actb* in both cDNA and genomic DNA (gDNA) (Supplementary Table S1). Agarose gel electrophoresis analysis revealed that circFat3 of the expected size was present in both cDNA and gDNA when amplified with convergent primers. However, it was only detected in cDNA when using divergent primers (Fig. 2A). In the control group, convergent primers of *Actb* yielded bands in both cDNA and gDNA, whereas no band was amplified using divergent primers of *Actb*. These results confirmed that circFat3 has a circular structure. Furthermore, after digestion of total RNA with RNase R, circFat3 remained detectable, whereas linear Fat3 was digested (Fig. 2B). The quantitative analysis further confirmed that circFat3 was more resistant to RNase R digestion than linear RNA (Fig. 2C). The results from the 0 active unit (U) group also suggested that the digestion procedure may influence expression levels. The structure of circFat3 was predicted using the CircPrimer software and sequence data. Notably, circFat3 possesses a unique back-splicing junction derived from exon 1 of *Fat3*. Sanger sequencing further validated that the splice site of circFat3 was consistent with the in silico analysis (Fig. 2D). Alignment with gene sequences from NCBI revealed that it includes a partial 5’ upstream region and coding sequences of exon 1.

### Determination of reference genes

**Fig. 3:**
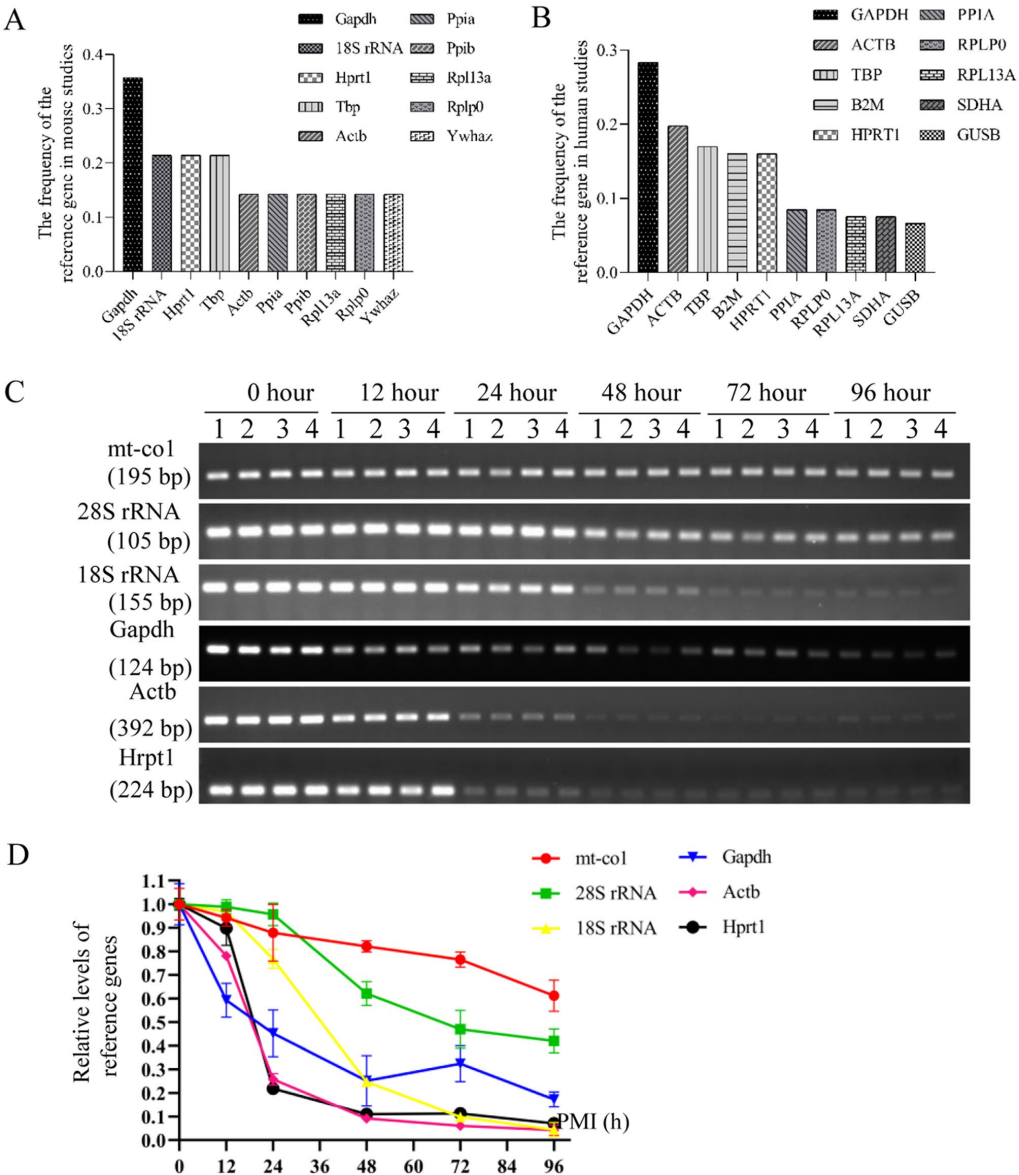
Selection of reference genes. (**A**) The usage frequencies of the top ten mRNAs in mouse studies from the ICG database. (**B**) The usage frequencies of the top ten mRNAs in human studies from the ICG database. (**C**) The postmortem levels of candidate reference genes in brain tissue at days 0, 1, 2, 4, and 8 at 35 °C. (**D**) The quantitative results of the relative expression in panel C.

According to the Internal control genes (ICG) database, the usage frequency of mRNAs in human and mouse studies was analyzed. The top 10 most frequently used mRNAs in mouse and human studies are shown in Fig. 3A,B, respectively. Then, the intersection of the five most commonly used reference genes in both humans and mice was selected for further analysis, including *Gapdh*, *Actb*, *Hprt1*, and *Tbp*. Additionally, mt-co1, 28S rRNA, and 18S rRNA were included as the reference genes. The stability of these seven candidate reference genes was evaluated by measuring their postmortem relative levels at different PMIs at 35℃. Electrophoresis analysis revealed that mt-co1 and 28S rRNA were more stable than the other markers (Fig. 3C). Notably, *Tbp* expression was so low that its bands were almost undetectable under the same amplification conditions (data not shown). The postmortem degradation trends of the reference genes were visualized in the line chart (Fig. 3D). The degradation rate of mRNAs was rapid, with none having a half-life exceeding 24 h. Among these markers, 28S rRNA was more stable than 18S rRNA and was deemed more suitable as a reference gene. Furthermore, mt-co1 showed the slowest degradation rate over time and remained relatively abundant even 96 h after death. Consequently, mt-co1 and 28S rRNA were selected as the reference genes for further study.

### Establishment and validation of the PMI estimation models at 4℃

**Fig. 4:**
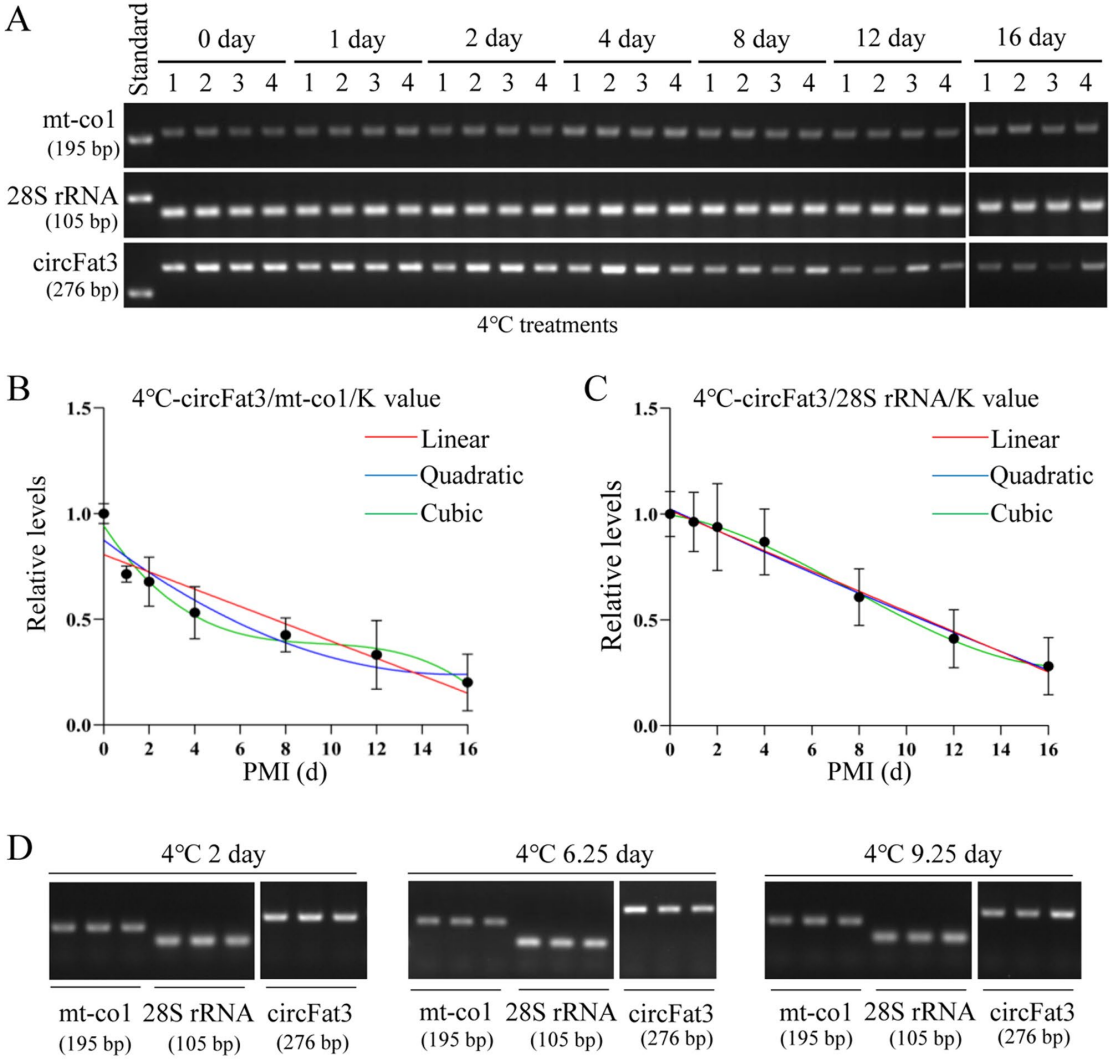
Construction and verification of 4 °C PMI models using semi-quantitative RT-PCR. (**A**) The postmortem levels of mt-co1, 28S rRNA, and circFat3 in brain tissue at the indicated days at 4 °C. (**B**) The linear, quadratic, and cubic equations constructed based on circFat3 and mt-co1 at 4 °C. (**C**) The linear, quadratic, and cubic equations constructed based on circFat3 and 28S rRNA at 4 °C. (**D**) The results of verification samples at days 2, 6.25, and 9.25 for 4 °C models. The grouping of gels cropped from different parts of the same gel was divided by the white space.

Total RNA was extracted from brain tissue samples at day 0, 1, 2, 4, 8, 12, and 16 postmortems at 4 °C. The results showed the postmortem levels of circFat3 and reference genes including mt-co1 and 28S rRNA (Fig. 4A). These results indicated that mt-co1 and 28S rRNA remained stable at low temperature, while circFat3 maintained a relatively stable level for up to 8 days postmortem. Based on the gray values of the bands, two unknown factors, such as relative levels and the K value, were calculated to build the mathematical models. In these models, the relative levels and PMI were assigned as the Y and X values, respectively. The ratio of the gray value of the biomarker to the gray value of the reference genes on day 0 was defined as the K value, which was 2.587 for circFat3/mt-co1 and 1.306 for circFat3/28S rRNA. The ratio of the gray value of the circFat3 to the gray values of the reference genes at different PMIs was calculated to construct the equations (Table 1), which are visualized in Fig. 4B,C. Based on the reference gene mt-co1, the R^2^ and *p* values for the linear, quadratic, and cubic equations were 0.8604 (*p* = 0.0026), 0.9208 (*p* = 0.0063), and 0.9724 (*p* = 0.0077), respectively. The R^2^ values of these equations were all greater than 0.86, indicating a good fit. Based on the reference gene 28S rRNA, the R^2^ and the *p* values for the three equations were 0.9900 (*p* < 0.0001), 0.9906 (*p* = 0.0001), and 0.9986 (*p* = 0.0001), respectively. Additionally, the equations were constructed using the combined application of two reference genes. Based on the two reference genes, the R^2^ values were all greater than 0.9700 and the *p* values were all less than 0.005. Based on the R^2^ and *p* values, the circFat3/28 s rRNA model may be more suitable for PMI estimation at the low temperature. Further details of the mathematical models are provided in Table 1.

**Table 1:** Establishment of the mathematical models for 4 °C PMI estimation by semi-quantitative RT-PCR.

Temperature	circRNA	Reference gene	Equation		R^2^	*P*
4 °C	circFat3	mt-co1	Linear	Y = -0.04098X + 0.8063	0.8604	0.0026
			Quadratic	Y = 0.8749–0.08176X + 0.002626X^2^	0.9208	0.0063
			Cubic	Y = 0.9429–0.1633X + 0.01646X^2^-0.0005742X^3^	0.9724	0.0077
		28S rRNA	Linear	Y = -0.04774X + 1.017	0.9900	0.0000
			Quadratic	Y = 1.025–0.05209X + 0.0002798X^2^	0.9906	0.0001
			Cubic	Y = 0.9955–0.01731X-0.005624X^2^ + 0.0002451X^3^	0.9986	0.0001
		mt-co1/28S rRNA	Linear	Y = -0.04436X + 0.9118	0.9705	0.0001
			Quadratic	Y = 0.9497–0.06693X + 0.001453X^2^	0.9883	0.0001
			Cubic	Y = 0.9692–0.09028X + 0.005417X^2^-0.0001646X^3^	0.9924	0.0011

The validation samples with known PMIs (2, 6.25, and 9.25 days) were used to test the accuracy of these mathematical models at 4℃ (Fig. 4D). In the circFat3/mt-co1 models, the error rates of 2nd-day samples, calculated by the linear, quadratic, and cubic equations were 225.50%, 143.05%, and 81.52%, respectively. For the 6.25th-day samples, the error rates of the linear, quadratic, and cubic equations were 14.22%, 14.73%, and 35.30%, respectively. For the 9.25th-day samples, the error rates of the three equations were 3.25%, 26.30%, and 38.04%, respectively. In the circFat3/28 s rRNA models, the error rates of the 2nd-day samples were 127.12%, 121.09%, and 148.95%, respectively. For the 6.25th-day samples, the error rates of the three equations were 1.24%, 1.50%, and 3.40%, respectively. For the 9.25th-day samples, the error rates of the three equations were 35.49%, 37.26%, and 33.29%, respectively.

When the combination of reference genes was used for the models, the error rates of the 2nd-day samples for the three equations were 276.75%, 223.49%, and 211.15%, respectively. The error rates of the 6.25th-day samples for the three equations were 28.96%, 11.34%, and 8.69%, respectively; while the error rates of the 9.25th-day samples for the three equations were 9.70%, 21.73%, and 22.94%, respectively (Table 2). The error rates of the 2nd-day samples were significantly higher than those of the 6.25th-day and 9.25th-day samples. The results showed that circFat3/28S rRNA models outperformed the others at 4℃. In addition, the joint application of reference genes did not significantly improve prediction accuracy and only contributed to long-term PMI estimation.

**Table 2:** Verification of the 4 °C mathematical models by semi-quantitative RT-PCR.

Temperature	circRNAs	Reference gene	Real PMI	Estimation PMI	Estimation Error	Error rate (%)	Equation
4 °C	circFat3	mt-co1	2d	6.5100	4.5100	225.50%	Linear
			2d	4.8609	2.8609	143.05%	Quadratic
			2d	3.6304	1.6304	81.52%	Cubic
			6.25d	7.1388	0.8888	14.22%	Linear
			6.25d	5.3294	0.9206	14.73%	Quadratic
			6.25d	4.0436	2.2064	35.30%	Cubic
			9.25d	8.9496	0.3004	3.25%	Linear
			9.25d	6.8177	2.4323	26.30%	Quadratic
			9.25d	5.7316	3.5184	38.04%	Cubic
		28S rRNA	2d	4.5425	2.5425	127.12%	Linear
			2d	4.4217	2.4217	121.09%	Quadratic
			2d	4.9791	2.9791	148.95%	Cubic
			6.25d	6.3275	0.0775	1.24%	Linear
			6.25d	6.1563	0.0937	1.50%	Quadratic
			6.25d	6.4622	0.2122	3.40%	Cubic
			9.25d	5.9675	3.2825	35.49%	Linear
			9.25d	5.8037	3.4463	37.26%	Quadratic
			9.25d	6.1710	3.0790	33.29%	Cubic
		mt-co1/28S rRNA	2d	7.5351	5.5351	276.75%	Linear
			2d	6.4697	4.4697	223.49%	Quadratic
			2d	6.2229	4.2229	211.15%	Cubic
			6.25d	8.0597	1.8097	28.96%	Linear
			6.25d	6.9590	0.7090	11.34%	Quadratic
			6.25d	6.7930	0.5430	8.69%	Cubic
			9.25d	8.3524	0.8976	9.70%	Linear
			9.25d	7.2398	2.0102	21.73%	Quadratic
			9.25d	7.1280	2.1220	22.94%	Cubic

### Establishment and validation of the 25℃ PMI estimation models

**Fig. 5:**
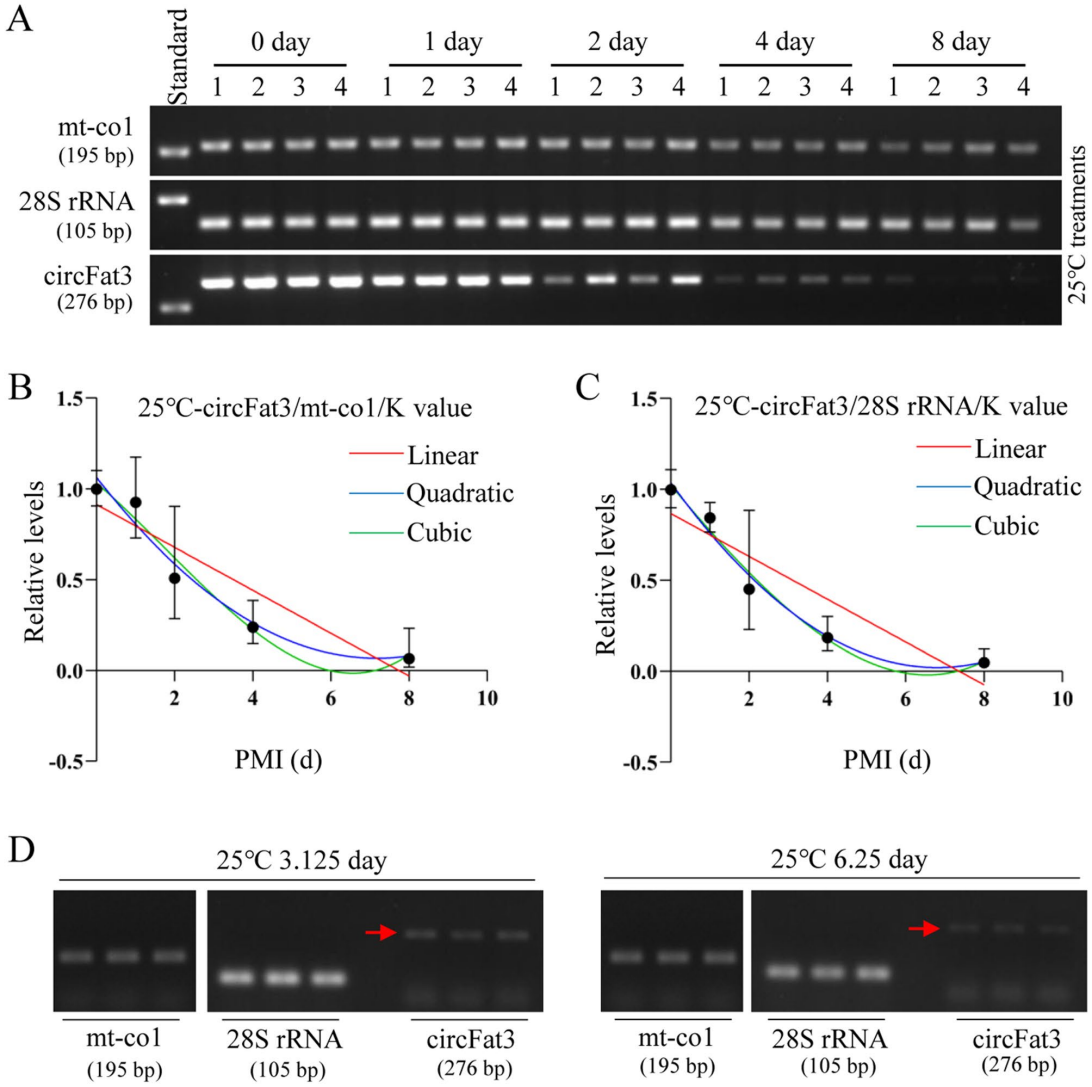
Construction and verification of 25 °C PMI models using semi-quantitative RT-PCR. (**A**) The postmortem levels of mt-co1, 28S rRNA, and circFat3 in brain tissue at the indicated days at 25 °C. (**B**) The linear, quadratic, and cubic equations constructed based on circFat3 and mt-co1 at 25 °C. (**C**) The linear, quadratic, and cubic equations constructed based on circFat3 and 28S rRNA at 25 °C. (**D**) The results of verification samples at 3.125 and 6.25 days for 25 °C models. The grouping of gels cropped from different gels was divided by the white space. The standard sample was used to eliminate the influence of exposure conditions.

Total RNA was extracted from brain tissue samples of sixteen-week-old mice at day 0, 1, 2, 4, and 8 postmortems at 25 °C. The amplification results of circFat3, mt-co1, and 28S rRNA were assessed by agarose gel electrophoresis (Fig. 5A). The results indicated that the degradation of all markers was faster than at low temperature, while mt-co1 and 28S rRNA remained stable at room temperature. CircFat3 maintained a relatively stable level for up to 1 day postmortem. At 25 °C, the K values of circFat3/mt-co1 and circFat3/28S rRNA were 2.226 and 1.962, respectively. The ratios of the gray value of circFat3 to the gray values of reference genes at different PMIs were calculated to construct the equations (Table 3), as shown in Fig. 5B,C. Based on the reference gene mt-co1, the R^2^ and the *p* values for the linear, quadratic, and cubic equations were 0.8495 (*p* = 0.0260), 0.9641 (*p* = 0.0359), and 0.9707 (*p* = 0.216), respectively. The R^2^ values of these equations were more than 0.84, indicating a good fit. Based on the reference gene 28S rRNA, the R^2^ and the *p* values of the three equations were 0.8371 (*p* = 0.0294), 0.9842 (*p* = 0.0158), and 0.9856 (*p* = 0.1523), respectively. Based on the joint application of two reference genes, the R^2^ and the *p* values for these equations were 0.8447 (*p* = 0.0273), 0.9753 (*p* = 0.0247), and 0.9788 (*p* = 0.1848), respectively. According to the R^2^ values, all cubic equations demonstrated a better fit than the corresponding linear and quadratic equations. However, the *p* values for all cubic equations were greater than 0.05. Further details about the mathematical models are provided in Table 3.

**Table 3:** Establishment of the mathematical models for 25 °C PMI estimation by semi-quantitative RT-PCR.

Temperature	circRNA	Reference gene	Equation		R^2^	*P*
25 °C	circFat3	Mt-co1	Linear	Y = -0.1181X + 0.9140	0.8495	0.0260
			Quadratic	Y = 1.064–0.2777X + 0.01934X^2^	0.9641	0.0359
			Cubic	Y = 1.033–0.1863X-0.01633X^2^ + 0.003105X^3^	0.9707	0.2169
		28S rRNA	Linear	Y = -0.1177X + 0.8671	0.8371	0.0294
			Quadratic	Y = 1.037–0.2990X + 0.02198X^2^	0.9842	0.0158
			Cubic	Y = 1.023–0.2566X + 0.005403X^2^ + 0.001443X^3^	0.9856	0.1523
		mt-co1/28S rRNA	Linear	Y = -0.1179X + 0.8906	0.8447	0.0273
			Quadratic	Y = 1.051–0.2884X + 0.02066X^2^	0.9753	0.0247
			Cubic	Y = 1.028–0.2215X-0.005465X^2^ + 0.002274X^3^	0.9788	0.1848

The validation samples with known PMIs (3.125 and 6.25 days) were used to test the accuracy of these mathematical models at 25℃ (Fig. 5D). In the circFat3/mt-co1 models, the error rates for the 3.125th-day samples calculated using the linear, quadratic, and cubic equations were 94.87%, 47.53%, and 33.94%, respectively. For the 6.25th-day samples, the error rates for the three equations were 14.56%, 10.78%, and 18.17%, respectively. In the circFat3/28 s rRNA models, the error rates for the 3.125th-day samples were 114.55%, 65.77%, and 52.83%, respectively. For the 6.25th-day samples, the error rates for the three equations were 12.39%, 6.49%, and 17.30%, respectively.

When the combination of reference genes was used for the models, the error rates for the 3.125th-day samples in the three equations were 104.71%, 55.88%, and 41.96%, respectively. For the 6.25th-day samples, the error rates for the three equations were 14.00%, 3.01%, and 17.21%, respectively (Table 4). The error rates for 3.125th-day samples were significantly higher than those of the 6.25th-day samples. The results showed that the circFat3/mt-co1 models were better than circFat3/28S rRNA models for short-term PMI estimation at 25℃. This finding indicated that the joint application of reference genes resulted in a more accurate long-term PMI estimation at 25℃.

**Table 4:** Verification of the 25 °C mathematical models by semi-quantitative RT-PCR.

Temperature	circRNAs	Reference gene	Real PMI	Estimation PMI	Estimation Error	Error rate (%)	Equation
25 °C	circFat3	Mt-co1	3.125d	6.09	2.96	94.87%	Linear
			3.125d	4.61	1.49	47.53%	Quadratic
			3.125d	4.19	1.06	33.94%	Cubic
			6.25d	7.16	0.91	14.56%	Linear
			6.25d	6.92	0.67	10.78%	Quadratic
			6.25d	5.11	1.14	18.17%	Cubic
		28S rRNA	3.125d	6.70	3.58	114.55%	Linear
			3.125d	5.18	2.06	65.77%	Quadratic
			3.125d	4.78	1.65	52.83%	Cubic
			6.25d	7.02	0.77	12.39%	Linear
			6.25d	5.84	0.41	6.49%	Quadratic
			6.25d	5.17	1.08	17.30%	Cubic
		mt-co1/28S rRNA	3.125d	6.40	3.27	104.71%	Linear
			3.125d	4.87	1.75	55.88%	Quadratic
			3.125d	4.44	1.31	41.96%	Cubic
			6.25d	7.12	0.87	14.00%	Linear
			6.25d	6.44	0.19	3.01%	Quadratic
			6.25d	5.17	1.08	17.21%	Cubic

Total RNA was extracted from brain tissue samples of nine-week-old mice at day 0, 1, 2, 4, and 8 postmortems at 25 °C. The amplification results of circFat3, mt-co1, and 28S rRNA were assessed by agarose gel electrophoresis (Supplementary Fig. S2A). It was observed that both circFat3 and the reference genes degraded more rapidly at 25 °C than at 4 °C. The K values of circFat3/mt-co1 and circFat3/28S rRNA were 2.742 and 1.657, respectively. The ratios of the gray value of circFat3 to the gray values of reference genes at different PMIs were calculated to construct the equations (Supplementary Table S2), as shown in Supplementary Fig. S2B,C. According to the R^2^ values, all cubic equations demonstrated a better fit than the corresponding linear and quadratic equations. However, the quadratic equations showed better *p* values. Further details about the mathematical models are provided in Supplementary Table S2. The validation samples with known PMIs (1.5 and 4 days) were used to test the accuracy of these mathematical models at 25℃ (Supplementary Fig. S2D). The results showed that the circFat3/mt-co1 models were better than circFat3/28S rRNA models for PMI estimation at 25℃. This finding indicated that the joint application of reference genes resulted in a more accurate long-term PMI estimation at 25℃ (Supplementary Table S3). It can be observed that the accuracy of the models established by mice of different ages is comparable.

### Establishment and validation of 35℃ PMI estimation models using semi-quantitative RT-PCR

**Fig. 6:**
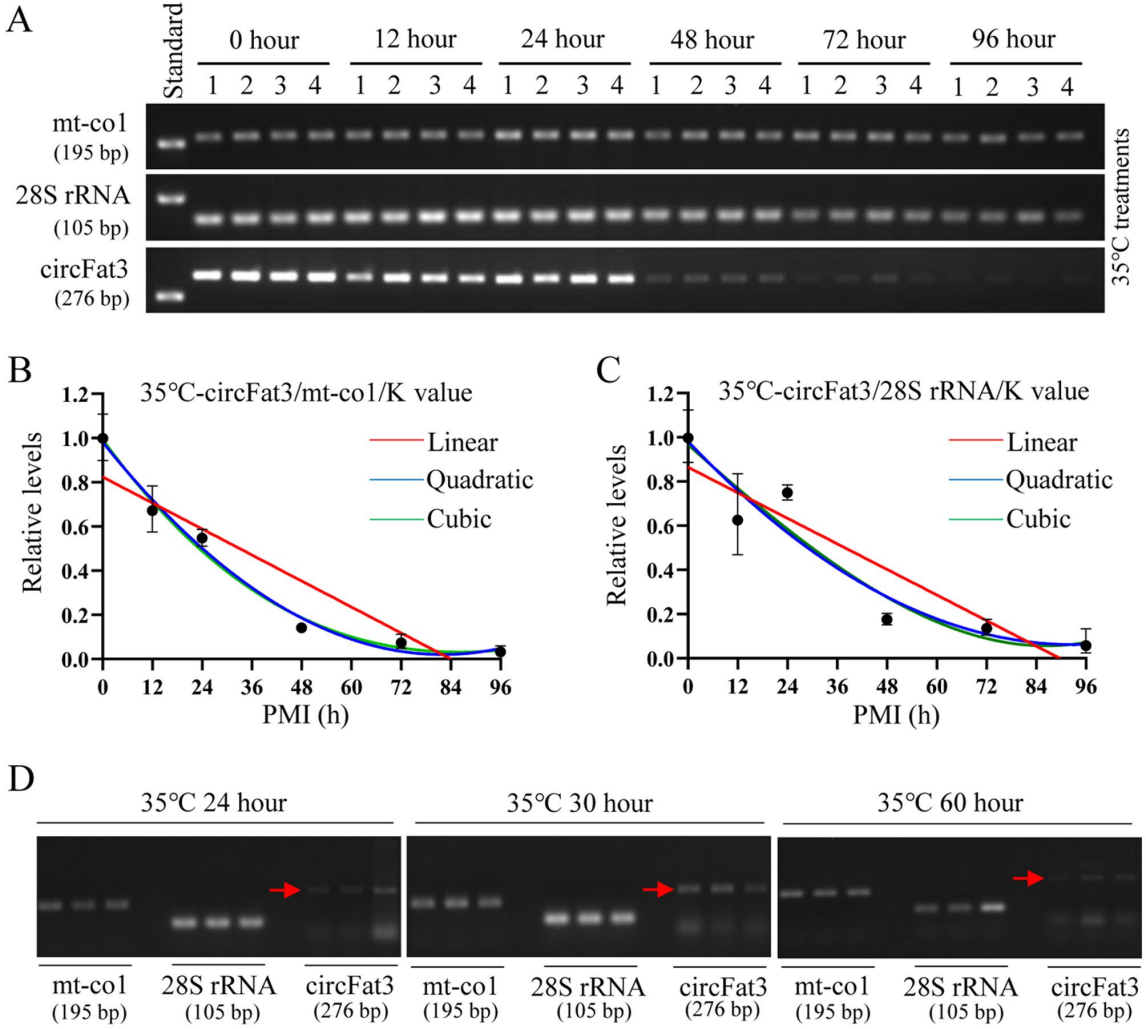
Construction and verification of 35 °C PMI models using semi-quantitative RT-PCR. (**A**) The postmortem levels of mt-co1, 28S rRNA, and circFat3 in brain tissue at the indicated hours at 35 °C. (**B**) The linear, quadratic, and cubic equations constructed based on circFat3 and mt-co1 at 35 °C. (**C**) The linear, quadratic, and cubic equations constructed based on circFat3 and 28S rRNA at 35 °C. (**D**) The results of verification samples at 24, 30, and 60 h for 35 °C models.

Total RNA from brain tissue samples was extracted at 0, 12, 24, 48, 72, and 96 h after death at 35 °C. The amplification results of circFat3, mt-co1, and 28S rRNA were analyzed by the agarose gel electrophoresis (Fig. 6A). The results indicated that the degradation of circFat3 was faster than that of the other temperatures, while mt-co1 and 28S rRNA remained stable at the high temperature. At 35 °C, the K values of circFat3/mt-co1 and circFat3/28S rRNA were 2.668 and 1.331, respectively. The ratio of the gray value of the circFat3 to the gray value of reference genes at different PMIs was calculated to construct the equations (Table 5), as shown in Fig. 6B,C. Based on the reference gene mt-co1, the R^2^ and the *p* values for the linear, quadratic, and cubic equations were 0.8639 (*p* = 0.0073), 0.9888 (*p* = 0.0012), and 0.9897 (*p* = 0.0154), respectively. The R^2^ values of these equations were all greater than 0.86, reflecting a good fit. Based on the reference gene 28S rRNA, the R^2^ and the *p* values for the three equations were 0.8496 (*p* = 0.0090), 0.9196 (*p* = 0.0228), and 0.9210 (*p* = 0.1162), respectively. Based on the joint application of two reference genes, the R^2^ and the *p* values of these equations were 0.8667 (*p* = 0.0070), 0.9634 (*p* = 0.0070), and 0.9634 (*p* = 0.0544), respectively. According to the R^2^ and *p* values, the circFat3/mt-co1 model may be more suitable for PMI estimation at the high temperature. In addition, all cubic equations showed a better fit than the corresponding linear and quadratic equations, but only the *p* values for the cubic equation in circFat3/mt-co1 models were less than 0.05. Further details about the mathematical models are provided in Table 5.

**Table 5:** Establishment of the mathematical models for 35 °C PMI estimation by semi-quantitative RT-PCR.

Temperature	circRNA	Reference gene	Equation		R^2^	*P*
35 °C	circFat3	mt-co1	Linear	Y = -0.009806X + 0.8240	0.8639	0.0073
			Quadratic	Y = 0.9813–0.02343X + 0.0001428X^2^	0.9888	0.0012
			Cubic	Y = 0.9933–0.02583X + 0.0002107X^2^-0.0000004698X^3^	0.9897	0.0154
		28S rRNA	Linear	Y = -0.009640X + 0.8650	0.8496	0.0090
			Quadratic	Y = 0.9818–0.01975X + 0.0001060X^2^	0.9196	0.0228
			Cubic	Y = 0.9672–0.01682X + 0.00002316X^2^ + 0.0000005735X^3^	0.9210	0.1162
		mt-co1/28S rRNA	Linear	Y = –0.009723X + 0.8445	0.8667	0.0070
			Quadratic	Y = 0.9816–0.02159X + 0.0001244X^2^	0.9634	0.0070
			Cubic	Y = 0.9803–0.02133X + 0.0001169X^2^ + 0.00000005185X^3^	0.9634	0.0544

The validation samples with known PMIs (24, 30, and 60 h) were used to test the accuracy of these mathematical models at 35℃ (Fig. 6D). In the circFat3/mt-co1 models, the error rates of 24th-hour samples calculated using the linear, quadratic, and cubic equations were 56.54%, 50.21%, and 51.75%, respectively. For the 30th-hour samples, the error rates for the three equations were 124.26%, 67.60%, and 68.93%, respectively. For the 60th-hour samples, the error rates for the three equations were 24.97%, 0.23%, and 3.69%, respectively. In the circFat3/28 s rRNA models, the error rates for the 24th-hour samples were 233.57%, 216.43%, and 195.56%, respectively. For the 60th-hour samples, the error rates for the three equations were 145.14%, 111.16%, and 103.37%, respectively. For the 60th-hour samples, the error rates for the three equations were 22.46%, 5.41%, and 1.55%, respectively.

When the combination of reference genes was used for the models, the error rates for the 24th-hour samples for the three equations were 213.54%, 164.15%, and 162.92%, respectively. The error rates of the 30th-hour samples for the three equations were 134.61%, 87.77%, and 87.26%, respectively, while the error rates for the 60th-hour samples were 23.73%, 2.93%, and 2.50%, respectively (Table 6). The error rates for the 60th-hour were significantly smaller than those for the 24th-hour and 30th-hour samples. The results showed that the circFat3/mt-co1 model performed better than the other models at 35℃. This finding suggested that the joint application of reference genes may only play a role in long-term PMI estimation.

**Table 6:** Verification of the 35 °C mathematical models by semi-quantitative RT-PCR.

Temperature	circRNAs	Reference gene	Real PMI	Estimation PMI	Estimation Error	Error rate (%)	Equation
35 °C	circFat3	mt-co1	24 h	10.43	13.57	56.54%	Linear
			24 h	11.95	12.05	50.21%	Quadratic
			24 h	11.58	12.42	51.75%	Cubic
			30 h	67.28	37.28	124.26%	Linear
			30 h	50.28	20.28	67.60%	Quadratic
			30 h	50.68	20.68	68.93%	Cubic
			60 h	74.98	14.98	24.97%	Linear
			60 h	60.14	0.14	0.23%	Quadratic
			60 h	62.21	2.21	3.69%	Cubic
		28S rRNA	24 h	80.06	56.06	233.57%	Linear
			24 h	75.94	51.94	216.43%	Quadratic
			24 h	70.93	46.93	195.56%	Cubic
			30 h	73.54	43.54	145.14%	Linear
			30 h	63.35	33.35	111.16%	Quadratic
			30 h	61.01	31.01	103.37%	Cubic
			60 h	73.48	13.48	22.46%	Linear
			60 h	63.25	3.25	5.41%	Quadratic
			60 h	60.93	0.93	1.55%	Cubic
		mt-co1/28S rRNA	24 h	75.25	51.25	213.54%	Linear
			24 h	63.40	39.40	164.15%	Quadratic
			24 h	63.10	39.10	162.92%	Cubic
			30 h	70.38	40.38	134.61%	Linear
			30 h	56.33	26.33	87.77%	Quadratic
			30 h	56.18	26.18	87.26%	Cubic
			60 h	74.24	14.24	23.73%	Linear
			60 h	61.76	1.76	2.93%	Quadratic
			60 h	61.50	1.50	2.50%	Cubic

### Establishment and validation of 35℃ PMI estimation models using RT-qPCR

**Fig. 7:**
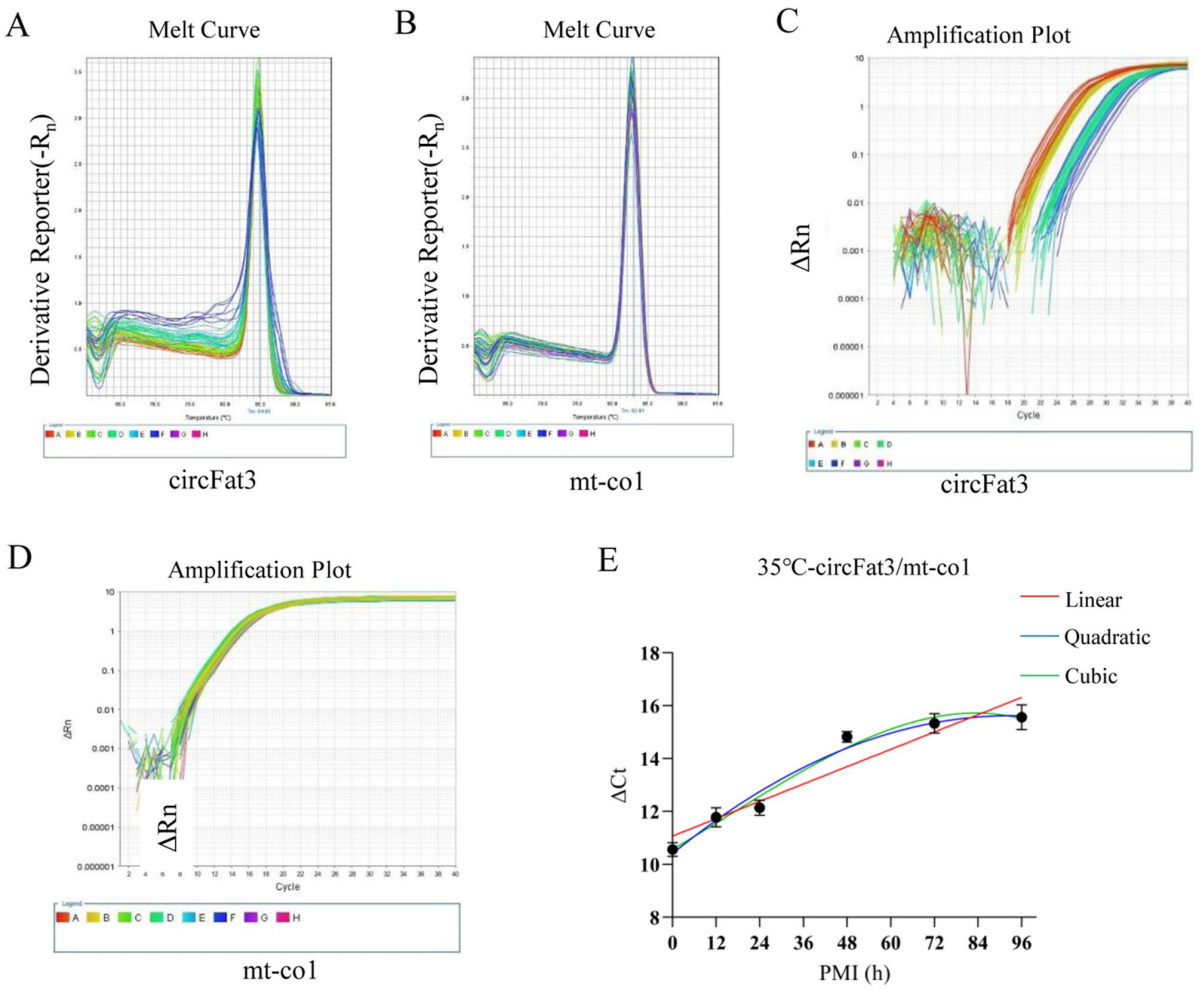
Construction and validation of 35 °C PMI models using RT-qPCR. (**A**) The melting curves of circFat3. (**B**) The melting curves of mt-co1. (**C**) The amplification curves of circFat3. (**D**) The amplification curves of mt-co1. (**E**) The linear, quadratic, and cubic equations constructed based on the ΔCt values of circFat3 and mt-co1 at 35 °C. h, hours.

The PMI estimation model at high temperature was further constructed based on RT-qPCR data. According to the results of semi-quantitative RT-PCR, circFat3 and mt-co1 were more suitable for PMI estimation at 35℃. The specificity of the primers was tested using fluorescence quantitative melting curves, which showed single peaks of both circFat3 and mt-co1, indicating that they were suitable for further experiments. This study presented the melt curve and amplification plot of circFat3 and mt-co1, demonstrating that the sample error was minimal and the data was reliable (Fig. 7A,B,C, and D). Based on the ΔCt values, the circFat3/mt-co1 models were constructed and visualized in Fig. 7E. The R^2^ and the *p* values of the linear, quadratic, and cubic equations were 0.8998 (*p* = 0.0039), 0.9740 (*p* = 0.0042), and 0.9797 (*p* = 0.0304), respectively (Table 7). The results showed that the data fit the equations well, and the fitted curve was reliable. Samples with known PMIs (24, 30, and 60 h) were also used to test the accuracy of these equations (Table 8). For the 24th-hour samples, the error rates for the linear, quadratic, and cubic equations were 100.10%, 51.99%, and 58.08%, respectively. For the 30th-hour samples, the error rates for the three equations were 53.56%, 16.43%, and 21.82%, respectively. For the 60th-hour samples, the error rates for the three equations were 32.02%, 22.95%, and 11.11%, respectively. The results revealed that the nonlinear equations were significantly more accurate than the linear equation. This RT-qPCR experiment further verified the semi-quantitative RT-PCR results, showing that these equations are better suited for PMI estimation over long periods of time.

**Table 7:** Establishment of the mathematical models for 35 °C PMI estimation by qPCR.

Temperature	circRNA	Reference gene	Equation		R^2^	*P*
35 °C	circFat3	mt-co1	Linear	Y = 0.05461X + 11.07	0.8998	0.0039
			Quadratic	Y = 10.41 + 0.1119X-0.0006007X^2^	0.9740	0.0042
			Cubic	Y = 10.57 + 0.07922X + 0.0003241X^2^-0.000006399X^3^	0.9797	0.0304

**Table 8:** Verification of the 35 °C mathematical models by qPCR.

Temperature	circRNAs	Reference gene	Real PMI	Estimation PMI	Estimation Error	Error rate (%)	Equation
35 °C	circFat3	mt-co1	24 h	48.02	24.02	100.10%	Linear
			24 h	36.48	12.48	51.99%	Quadratic
			24 h	37.94	13.94	58.08%	Cubic
			30 h	46.07	16.07	53.56%	Linear
			30 h	34.93	4.93	16.43%	Quadratic
			30 h	36.55	6.55	21.82%	Cubic
			60 h	79.21	19.21	32.02%	Linear
			60 h	73.77	13.77	22.95%	Quadratic
			60 h	66.67	6.67	11.11%	Cubic

## Discussion

PMI refers to the time from the discovery of the remains to the time of death. PMI estimation plays an important role in forensic medicine, including case investigation and civil actions. In recent decades, forensic scientists have mainly inferred the time of death based on various changes in the body after death such as algor mortis, livor mortis, and rigor mortis. These methods are often influenced by various intrinsic and extrinsic factors and are prone to producing false positives or inaccurate results. With advances in molecular biology, the time-dependent degradation of biological markers (e.g. proteins, DNA, and RNA) has become a focal point in PMI estimation. The characteristics of circRNA, such as its conservation, high abundance, stability, and specificity, make it a promising candidate for PMI estimation^15,28^. However, few studies have explored the use of circRNA for PMI estimation, and this area remains in an exploratory stage.

Recent studies have demonstrated that circRNAs play important roles in regulating cell death processes such as autophagy, apoptosis, and ferroptosis. Certain circRNAs involved in apoptosis have been linked to cancer development and can influence prognosis^29^. Moreover, circRNA-mediated autophagy can impact tumor progression and alter chemoresistance in various types of cancer^30^. In ferroptosis, some circRNAs regulate ferroptosis-associated genes by sponging specific miRNAs^31^. As a class of non-coding RNAs involved in infraction, circRNAs have been extensively studied in myocardial infarction. However, research on circRNAs in cerebral infarction remains in its early stages. Current studies suggest that circRNAs may inhibit apoptosis following cerebral infarction^32^. In our team’s previous research, we identified circRnf169 in liver tissue as a potential biomarker for early PMI estimation^33^. However, due to the rapid degradation of circRNAs in liver tissue, it is challenging to use for late PMI estimation. Since circRNAs are conserved and abundant in brain tissue^34^, in this study, circRNAs with specificity in the brain tissue were the primary focus. In addition, temperature is a major extrinsic factor that affects decomposition after death^35^. Therefore, it is crucial to study the degradation of biomarkers after death while controlling for temperature as a variable.

This study found a strong association between circFat3 degradation levels at multiple temperatures and PMI. Therefore, circFat3 was identified as a biomarker in brain tissue for constructing PMI estimation models. As a protein-coding gene, *FAT3* plays a crucial role in spinal cord development and retina layer formation^36^ and is related to a variety of cancers. Research has shown that circFat3 is abundant in brain tissue and conserved in both humans and mice^24^. Seeler et al. indicated that circFat3 plays an important role in neural development^37^. Hansen et al. demonstrated its potential as a biomarker in prostate cancer. Jiang et al. demonstrated its role in promoting lung cancer development^38^. These studies suggest that circFat3 is involved in multiple physiological and pathological processes. As one of the circular transcripts of *Fat3*, mmu_circ_0001746 was confirmed in our study to be formed by back-splicing of exon 1 of the *Fat3*. This circular transcript is conserved between humans and mice and exhibits high specificity in brain tissue. It has the potential for use in modeling and validation in human samples.

In addition, identifying an ideal internal reference gene is crucial for PMI estimation. Such a gene should exhibit stable degradation levels across different PMIs. Therefore, reference genes must be carefully selected when constructing the PMI estimation model. Tu et al.^39^ evaluated the stability of tissue-specific reference genes, particularly circRNAs, using the geNorm and NormFinder algorithms. Suitable internal reference genes were identified for PMI estimation from 11 candidates, including circ-AFF1. CircAFF1 was detected in our pre-experiments but was not further investigated due to its low expression in brain tissue. Our results indicate that postmortem mRNA degradation occurs rapidly, making mRNA unsuitable as a reference gene for the PMI estimation. In this study, mt-co1 was used for the first time as the reference gene in the brain tissue for PMI estimation. The results demonstrated that mt-co1 is a stable reference gene that can be applied to PMI estimation models.

This study demonstrated that the degradation pattern of circFat3 in brain tissue at multiple temperatures was closely associated with PMI. At different temperatures, the postmortem degradation trends of mt-co1 and 28S rRNA remained stable across various PMIs. Based on these markers, PMI estimation models were constructed using mouse brain tissue at low, room, and high temperatures. Notably, the K values of circFat3/mt-co1 and circFat3/28S rRNA varied across temperatures. The K values of nine-week-old mice at 4 °C, 25 °C and 35 °C were similar, whereas those of sixteen-week-old mice at 25 °C differed significantly from the others. This discrepancy may be due to the fact that the brain tissue samples for 25 °C PMI estimation were collected from the age of sixteen-week-old mice. The results suggested that age may influence the relative expression levels of circFat3, consistent with previous studies indicating that circRNA expression in neurodevelopment is age-dependent^40,41^. Of course, future studies should be conducted.

At low temperature, 28S rRNA proved to be a more suitable reference gene for the PMI estimation model. The R^2^ and *p* values of these models indicated good fit and reliability. However, these models tended to overestimate PMI for shorter times after death and underestimate PMI for longer ones. Notably, they demonstrated greater accuracy in long-term PMI estimation. Although error rates were high in the early postmortem period, the estimation errors at later time points were comparable to those of the early ones. Additionally, combining reference genes provided only a slight improvement in accuracy for long-term PMI estimation. At room temperature, the *p* values of these cubic equations exceeded 0.05, likely reflecting the complexity of the model. Thus, it is necessary to synthesize the validation results. Notably, the cubic equations demonstrated greater accuracy for short-term PMI estimation at 25 °C. Although circFat/mt-co1 and circFat/28S rRNA models exhibited similar accuracy for PMI estimation at 25 °C, the former had higher accuracy for the relatively short-time PMI estimation. In addition, combining reference genes provided only a modest improvement in accuracy for long-term PMI estimation. At high temperature, mt-co1 emerged as the more suitable reference gene for PMI estimation. The combination of reference genes was beneficial only for long-term PMI estimation at 35 °C. Nonlinear equations were found to be more accurate than linear ones based on RT-qPCR data. However, the joint application of reference genes did not substantially enhance predictive accuracy, indicating that model performance largely depended on selecting the most suitable reference gene. Therefore, it is essential to identify more suitable reference genes for these biomarkers.

This study pioneered the exploration of circRNA in brain tissue for PMI estimation. However, due to the lack of validation using human samples, such as the marker hsa_circ_0000348, its practical applicability requires further investigation. Future studies should explore additional circRNAs that are highly expressed across multi-tissue for PMI estimation. The combined application of multiple circRNAs, either within a single tissue or across different tissues, is likely to improve PMI estimation accuracy.

In conclusion, this research successfully constructed the PMI mathematical models at various temperatures using mouse samples. CircFat3 (mmu_circ_0001746) was identified as a potential biomarker in brain tissue for PMI estimation. The mt-co1 and 28S rRNA were found to be stable across multiple temperatures and suitable as reference genes for the models. The combined application of the two reference genes was beneficial primarily for long-term PMI estimation. These models demonstrated greater accuracy in the estimation of long-term PMI. In summary, this study may provide a novel approach for the estimation of PMI. However, several challenges should be addressed before this approach can be translated into forensic application, including the applicability of the models to human samples, the combined use of multiple tissues and markers, and the influence of factors such as microbial activity, pH, and humidity on postmortem degradation of circRNA.

## Materials and methods

### Human-mouse homologous circRNA selection

The existing circRNA databases such as circBase (http://circbase.org/) (accessed on Aug. 25, 2023)^42^ and circBank (http://www.circbank.cn/) (accessed on Aug. 25, 2023)^43^, as well as literature including the sequencing data of circRNA for human and mouse were used to screen for circRNAs^24^. First, circRNAs with high expression or strong tissue specificity in human and mouse brain were identified from the relevant literature. Then, the corresponding circRNAs were searched in circBase, and their circBase IDs were used to obtain the relevant information about human-mouse homologous circRNAs from circBank, including RNA sequence, genome position, length, and host gene symbol. The screened circular transcripts were queried in the GTEx (https://www.gtexportal.org/home/index.html) (accessed on May 25, 2024) and NCBI (https://www.ncbi.nlm.nih.gov/gene/) databases (accessed on May 25, 2024)^44^ to assess the expression of host genes across multiple tissues in humans and mice, respectively. The chromosomal location information of the circRNA was used to query the circAtlas (https://ngdc.cncb.ac.cn/circatlas/index.php) database (accessed on May 25, 2024)^45^ for the expression of the circular transcript in different tissues of humans and mice.

### Primer design of circRNA

Primers were designed based on the sequences on either side of the circRNA splice site. The convergent primer targeted the linear transcript of the mRNA corresponding to the circRNA, while the divergent primer was designed based on the back-spliced sequence, which usually combines a sequence at the 3’ end of the linear transcript with the upstream sequence at the 5’ end to form a sequence containing the splicing site. These primers were designed by primer 3.0 (https://bioinfo.ut.ee/primer3-0.4.0/) (accessed on Jun. 19, 2024) and NCBI blast (https://blast.ncbi.nlm.nih.gov/Blast.cgi) (accessed on Jun. 19, 2024) and their specificity was validated using the Primer-BLAST tool (https://www.ncbi.nlm.nih.gov/tools/primer-blast/) (accessed on Jun. 19, 2024). CircPrimer 2.0 software (Center of Clinical Laboratory Science, The Affiliated Cancer Hospital of Nanjing Medical University, China) was used to validate the specificity of the divergent primer and to illustrate its approximate position within the circRNA^46^. The details of these primers are listed in Supplementary Table S1.

### Preparation of mouse samples

A total of 36 male BALB/c mice (aged 9 weeks and 16 weeks, with body weight ranging from 23 to 26 g) of specific pathogen-free grade were purchased from Tengxin Biotechnology Co., Ltd. (Chongqing, China). The mice were humanely sacrificed after receiving an intraperitoneal injection of 50 mg per kilogram (mg/kg) of pentobarbital sodium for anesthesia in our laboratory, following the AVMA Guidelines for the Euthanasia of Animals (2020 edition) and the National Standards of the People’s Republic of China “Laboratory animal-Guidelines for euthanasia (GB/T 39760-2021)”. Animal experiments in this study were performed in accordance with the ARRIVE guidelines 2.0, and approved by the animal ethics committees of Southwest Medical University, with the approval number: 20230821–011. After euthanasia, the mouse samples were placed in a controlled temperature environment. Mice were grouped according to the different temperatures, including 4℃, 25℃, and 35℃, and brain tissues were collected at different PMIs. Specifically, at 4 °C, the brain tissues were extracted at day 0, 1, 2, 4, 8, 12, and 16 after death; at 25 °C, the brain tissues were extracted at day 0, 1, 2, 4, and 8 after death; and at 35 °C, the brain tissues were extracted at 0, 12, 24, 48, 72, and 96 h after death. These conditions are based on published literature and our preliminary study^6,33,39^. The normal rectal temperature of healthy mice ranges from 36.5 to 37.5 °C. Under the same temperature conditions, samples from five mice were collected at each PMI. After preliminary testing, four samples showing less variation at each PMI were selected for further study. The corresponding tissues were also collected at additional known PMIs to serve as validation samples. Known PMIs refer to samples collected at specific time points after death, which are used to validate the previously constructed PMI estimation models. These samples are distinct from those used to build the aforementioned models. In addition, 11 types of mouse tissues were sampled to assess circRNA expression. All mouse experiments followed the Instructive Notions with Respect to Caring for Laboratory Animals and Regulations for the administration of affairs concerning experimental animals in China.

### Total RNA and genomic DNA isolation

Total RNA was extracted from approximately 40 mg of brain tissue using the RNAsimple Total RNA Kit (Cat. #: DP210831, Tiangen Biotech Co., Ltd, Beijing, China). The experimental protocols were according to the manufacturer’s instructions. After the extraction of total RNA, the NanoDrop™ 2000 spectrophotometer (Thermo Fisher Scientific Inc, Waltham, USA) was used to check its concentration and purity. If the value of A260/A280 is between 1.8 and 2.0 and the value of A260/A230 is greater than 2.0, it indicates that the purity of total RNA is good. Certainly, RNA concentration also affects these values, particularly in degraded samples. For the integrity of total RNA, the 1% agarose gel electrophoresis was used to evaluate the 5S rRNA, 18S rRNA, and 28S rRNA bands. The extracted total RNA was stored directly at -80℃ or reverse transcribed into cDNA and stored at -20℃.

The gDNA was extracted from fresh tissues by the Phenol–chloroform Method^47^. The fresh tissue was ground with a small grinding stick in a new 1.5 mL Eppendorf tube (EP tube), followed by addition of 700 µL of Nucleic Lysis Buffer (0.001 mol/L Tris, 0.04 mol/L, 0.0002 mol/L EDTA (pH 8.0)), 80 µL of 20% SDS (Cat. #: A600848-0100, BBI Life Sciences Corporation, Shanghai, China) and 7 µL of proteinase K (20 mg/mL) (Cat. #:1245680100, Merck KGaA, Darmstadt, Germany) were added. The mixture was shaken and then centrifuged briefly. The sample was incubated at 56 °C for 4 h, during which it was vigorously shaken by hand at half-hour intervals. After the water bath, an equal volume of Tris balanced phenol (Cat. #: DP210831, Beijing Solarbio Science & Technology Co., Ltd., Beijing, China) was added to the tube and vortexed for several seconds. The sample was centrifuged for 5 min at 12,000 rpm and the colorless upper aqueous phase was carefully pipetted into a new 1.5 mL EP tube. Then, an equal volume of phenol/chloroform (V: V = 1:1) was added into the tube and shaken vigorously before centrifugation at 12,000 rpm for 5 min. The supernatant was pipetted into another 1.5 mL EP tube and 2.5 times the volume of absolute ethanol was added to the solution. The white flocculent precipitates appeared after thoroughly mixing and centrifugation at 12,000 rpm for 5 min. The supernatant was removed and the pellet was washed twice using 70% ethanol, followed by centrifugation at 12,000 rpm for 1 min each time. The DNA precipitate was dried at room temperature, and the ddH_2_O was added to dissolve it. The gDNA was stored at -20℃ for downstream experiments.

### Reverse transcription

Total RNA (1 µg) was reverse transcribed into cDNA using the ReverTra Ace™ qPCR RT Master Mix (Cat. #: FSQ-201, TOYOBO CO., LTD., Osaka, OS, Japan). The total reaction volume was 10 μL and all reagents, including 5 × RT Master Mix, RNA template, and Nuclease-free Water, were prepared on ice. The mixed reagents were then incubated at 37 °C for 15 min, followed by 50 °C for 5 min, and then heated to 98 °C for 5 min. This resulting solution was stored at -20℃ or diluted tenfold for subsequent experiments.

### Validation of circRNA

The convergent and divergent primers for different circRNAs and the control gene *Actb* were designed (Supplementary Table S1). PCR products were analyzed by the agarose gel electrophoresis. The convergent and divergent primers were used to test these circRNAs and *Actb* in cDNA and gDNA. In cDNA, the bands amplified by divergent primers showed distinct sizes compared to those obtained with convergent primers, whereas no amplification was observed with divergent primers in gDNA, confirming that circRNA undergoes back-splicing to form a circular structure.

In addition, the RNase R (Cat. #: R0300; Guangzhou Geneseed Biotech Co., LTD., Guangzhou, China) was used to treat the samples for detecting the expression of circRNA and its corresponding linear RNA. RNase R, an exonuclease derived from *Escherichia coli*, digests nearly all linear RNA molecules but has difficulty digesting circRNA. The RNase R digestion assay includes the following steps. For each sample, 200 nanograms (ng) of total RNA was used for RNase R digestion. The control group received 1 μL of 10 × Reaction Buffer and was adjusted to a final volume of 10 μL with RNase-Free Water. For the first treatment group (0 U), 1 μL of 10 × Reaction Buffer was added to the mixture. For the second treated group (1 U), 1 U of RNase R (1U/μL) and 1 μL of 10 × Reaction Buffer were added. Both treatment groups underwent enzymatic digestion at 37℃ for 15 min followed by enzyme inactivation at 70℃ for 10 min.

Sanger sequencing was applied to confirm the splice junction of circRNA for definitive validation. The sequencing of PCR products amplified by divergent primers was performed using a 3500Dx Genetic Analyzer (Applied Biosystems, Life Technology, Carlsbad, USA).

### Identification of reference genes

The ICG (https://ngdc.cncb.ac.cn/icg/) (accessed on Oct. 1, 2023) is a curated database of reference genes for humans and mice^48^. Based on a literature review, the ICG database has a comprehensive collection of high-quality reference genes. Consequently, several mRNAs commonly used as reference genes in studies of humans and mice were initially screened. The intersection of the top five reference genes was selected as candidate markers. Moreover, 28S rRNA and 18S rRNA were selected as the candidate reference genes, as they are often used for PMI estimation. In addition, cytochrome c oxidase subunit I (mt-co1), a mitochondrial mRNA encoded by mitochondrial DNA, is primarily expressed based on its half-lives and is resistant to RNases^49^. Thus, mt-co1 has the potential to serve as a reference gene. For this purpose, a total of seven markers were screened as candidate reference genes. Finally, high-temperature samples were used to investigate the degradation of candidate reference genes after death using semi-quantitative RT-PCR.

### The detection of circRNA degradation level

Semi-quantitative RT-PCR and RT-qPCR were applied to assess the levels of these circRNAs in different PMIs at multiple temperatures. Initially, the semi-quantitative RT-PCR was used to detect the level of circRNA degradation. The total reaction volume was 10 μL, including 5 μL of 2 × Taq PCR Master Mix (Cat. #: DP210831, Tiangen Biotech Co., Ltd, Beijing, China), 3 μL of RNase-Free Water, 1 μL of divergent primer pairs, and 1 μL of cDNA. For circRNA biomarkers, the PCR procedure was set as follows: the first step of pre-denaturation at 95℃/90 s; 30 cycles of the second step of denaturation at 95℃/30 s, annealing at 65℃/30 s, extension at 72℃/25 s; the third step of final extension at 72℃/5 min. For reference genes, the second step of the PCR procedure differed from that of the circRNA markers. It was set as follows: denaturation at 95℃/30 s, annealing at 60℃/30 s, extension at 72℃/25 s, and 19 cycles for mt-co1 or 20 cycles for 28S rRNA. These amplification reactions were performed using an Applied Biosystems Veriti® 96-Well Thermal Cycler PCR (Thermo Fisher Scientific Inc, Waltham, USA). After PCR amplification, a 1.5% agarose gel was utilized for electrophoresis, and exposure imaging was performed using a Bio-Rad Universal Hood II (Bio-Rad Laboratories, Inc, State of Delaware, USA).

For the RT-qPCR, the 2 × Fast SYBR Green qPCR Master Mix Kit (Cat. #:3325–05, Servicebio Technology Co., Ltd., Wuhan, China) was used to perform the quantitative analysis of circRNA expression. According to the manufacturer’s instructions, a reaction mix (10 μL) was prepared, consisting of 3 μL of RNase-Free Water, 1 μL of divergent primer pairs, 1 μL of cDNA, and 5 μL of 2 × Fast SYBR Green qPCR Master Mix (High ROX). This experiment was performed using an Applied Biosystems StepOneplus™ Real-Time PCR System (Thermo Fisher Scientific Inc, Waltham, USA). The cycling conditions were established as follows: the first step of PCR initial heat activation at 95℃/2 min; 40 cycles of the second step of denaturation at 95℃/5 s and combined annealing/extension at 60℃/20 s; and the third step of melting curve analysis at 95℃/15 s, 60℃/1 min, and 95℃/15 s. The samples were tested in triplicate and analyzed only when the standard deviation was less than 0.2.

### Construction of standard samples

Standard samples were used to eliminate the influence of exposure conditions. Using mouse *Gapdh* primers, gDNA from the brain sample was amplified by PCR to generate the standard sample. The total volume of the reaction mixture was 100 μL, consisting of 50 μL of 2 × Taq PCR Master Mix (Cat. #: DP210831, Tiangen Biotech Co., Ltd, Beijing, China), 37.5 μL of RNase-Free Water, 10 μL of primer pairs, and 2.5 μL of gDNA (600 ng). The PCR procedure was set as follows: the first step of pre-denaturation at 95℃/90 s; 28 cycles of the second step of denaturation at 95℃/30 s, annealing at 60℃/30 s, extension at 72℃/25 s; and the third step of final extension at 72℃/5 min.

### Establishment and validation of PMI mathematical models

For the results of semi-quantitative RT-PCR, the ImageJ 1.54d (National Institutes of Health, Bethesda, USA) software was performed to test the integrated density (IntDen) values, which represent the grey value of bands. To remove the influence of the image background, the grey value of the background was subtracted from each band’s grey value. To eliminate the influence of exposure conditions, the aforementioned standard samples were used. In agarose gel analysis, an equal volume (3 μL) of the standard sample was used to normalize the gray value between circFat3 and reference genes. The normalization factor (M) was calculated by the following formula:

1 $${\text{M}} \, =\frac{\text{the background-subtracted grey value of the standard sample in the circFat3 agarose panels}}{\text{the background-subtracted grey value of the standard sample in the reference gene panels}}$$

After background subtraction, the average grey value of circRNA markers was divided by the average gray value of the reference genes at the same PMIs. This ratio represented the relative levels of circFat3 at different PMIs. In addition, a constant (K) was constructed for the PMI estimation model. The formulas were as follows:

2 $${\text{K}} \, = \, \frac{\text{M }\times \text{ (the }\text{background-subtracted} \, \text{gray value of circ}{\text{Fat3}}\text{ at 0-day}\text{)}}{\text{the }\text{background-subtracted}\text{ gray value of reference gene at 0-day}}$$

3 $$\text{Relative levels} \, = \, \frac{\text{M }\times \, \text{(the background-subtracted gray value of circ}{\text{Fat3}}\text{ at N-PMI)}}{\text{(the background-subtracted gray value of reference genes at N-PMI)} \, \times\text{ K}}$$

where N is the time since death.

For the results of RT-qPCR, the average value of reliable data about circRNAs and reference genes was used to calculate the ΔCt values in every PMI. The formulas were as follows:

4 $$\Delta Ct = {\text{ Ct }}\left( {{\text{circFat3}}} \right) - {\text{ Ct }}\left( {{\text{mt-co1}}} \right)$$

The IBM SPSS Statistics 25 (International Business Machines Co., Armonk, USA) software was utilized to build mathematical models and calculate the corresponding *p* value and R^2^ for linear, quadratic, and cubic regression equations. GraphPad Prism 8 (GraphPad Software, LLC., San Diego, USA) was used to display the regression curve for the linear and nonlinear equations.

The accuracy of PMI mathematical models was validated by calculating the error rate, which was determined using the following formula:

5 $$\text{Error rate}=\frac{|\text{ (estimated PMI }-\text{ real PMI) }|}{\text{real PMI}} \,\times \text{100}\%$$

## Electronic supplementary material

Below is the link to the electronic supplementary material.

Supplementary Material 1

Supplementary Material 2

Supplementary Material 3

## Data Availability

Data is provided within the manuscript or supplementary information files.

## Declarations

### ARRIVE guidelines

All animal experiments were performed in accordance with ARRIVE 2.0 guidelines.
