# Supplementary material for: Circular RNA circFat3 as a biomarker for construction of postmortem interval Estimation models in mouse brain tissues at multiple temperatures
Source: Sci Rep. 2025 Jul 1;15:21577. doi: 10.1038/s41598-025-07998-0 (PMC12216475; doi:10.1038/s41598-025-07998-0)
Supplement: Supplementary file 1 — Supplementary Material 1 [file 41598_2025_7998_MOESM1_ESM.pptx]

## Slide 1
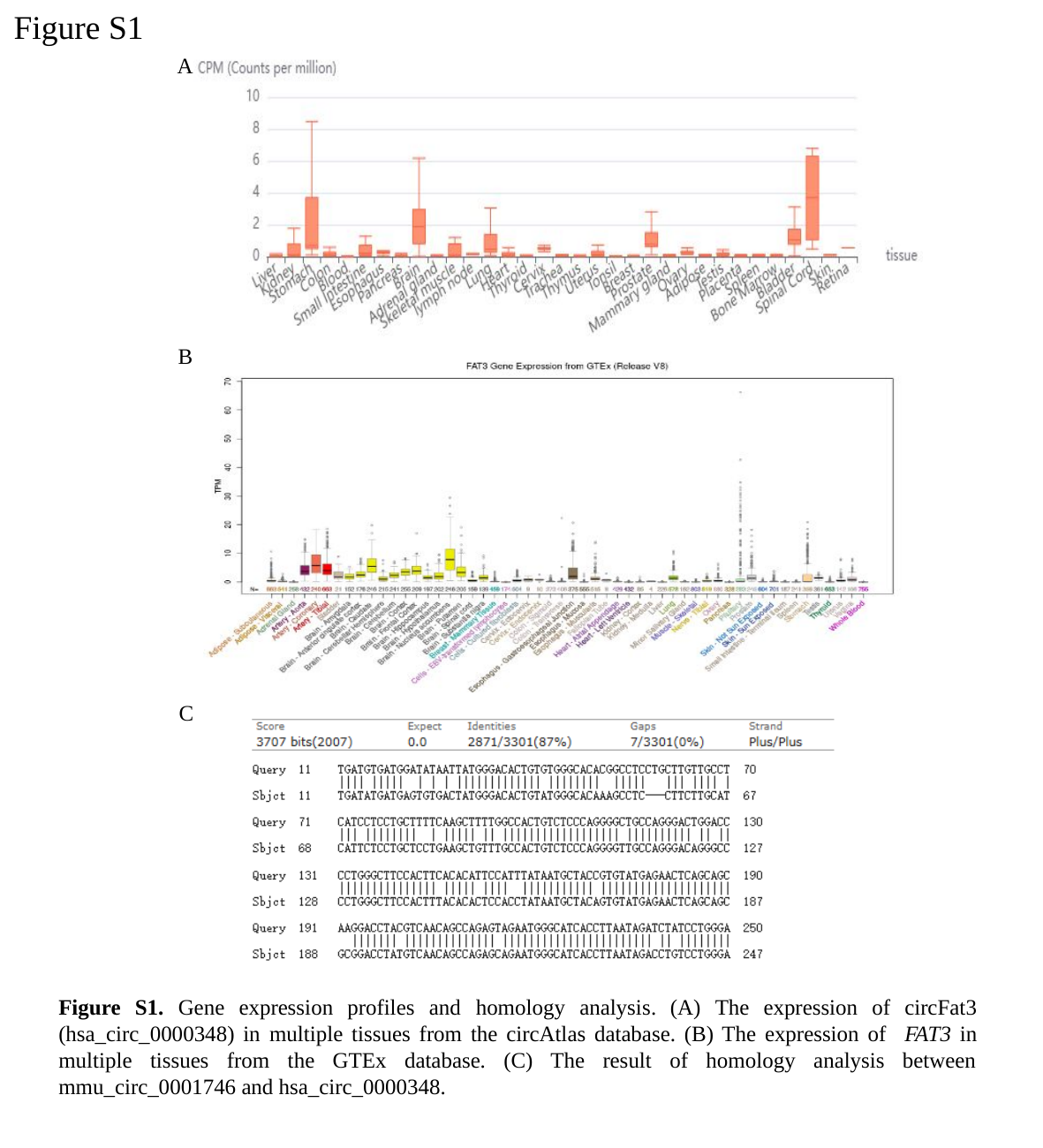

Figure S1
A
B
C
Figure S1. Gene expression profiles and homology analysis. (A) The expression of circFat3 (hsa_circ_0000348) in multiple tissues from the circAtlas database. (B) The expression of FAT3 in multiple tissues from the GTEx database. (C) The result of homology analysis between mmu_circ_0001746 and hsa_circ_0000348.

## Slide 2
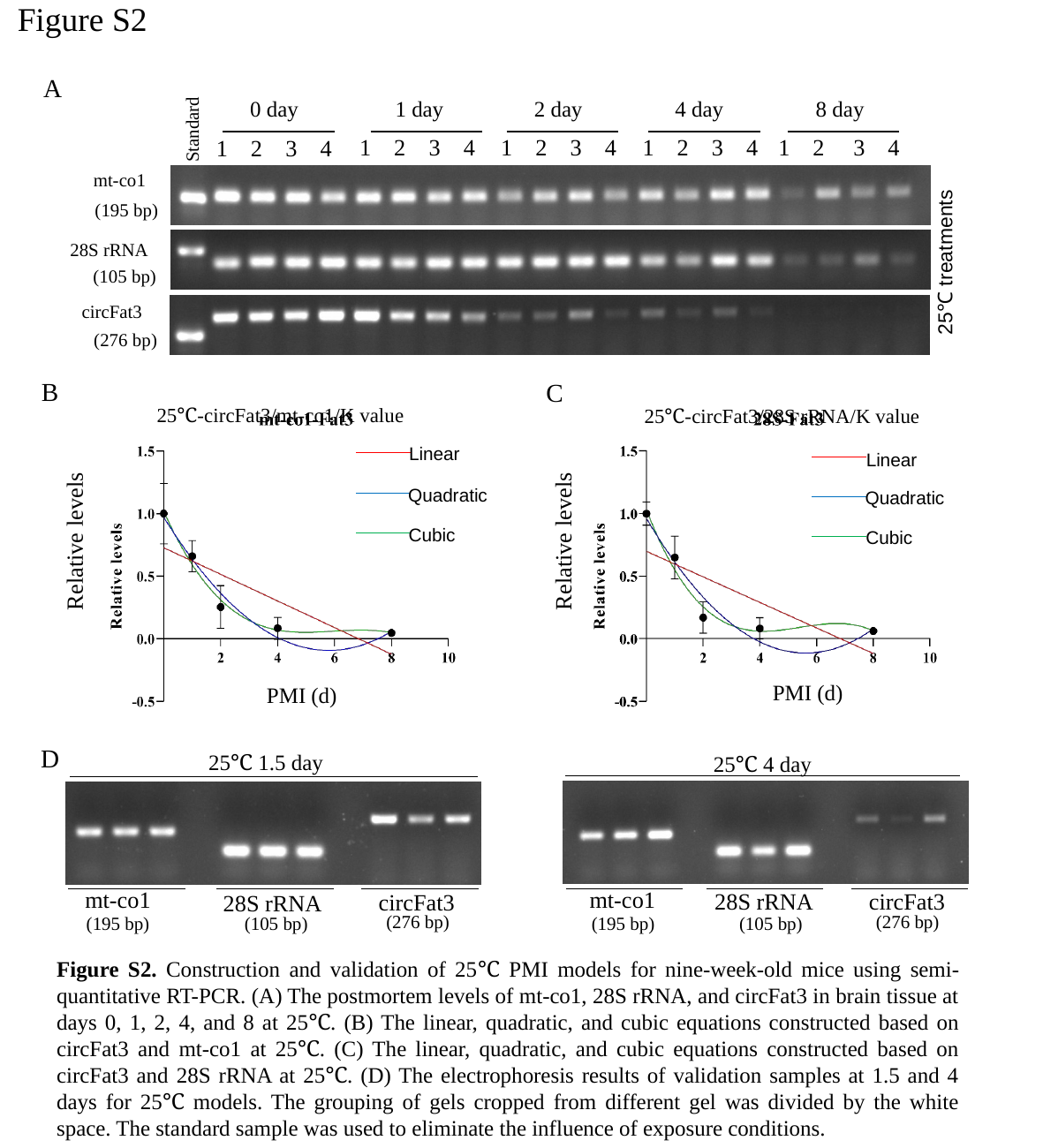

Figure S2
A
4 day
8 day
1 day
2 day
0 day
Standard
 1 2 3 4
1 2 3 4
1 2 3 4
 1 2 3 4
 1 2 3 4
mt-co1
(195 bp)
28S rRNA
25℃ treatments
(105 bp)
circFat3
(276 bp)
B
C
25℃-circFat3/mt-co1/K value
25℃-circFat3/28S rRNA/K value
Linear
Quadratic
Cubic
Linear
Quadratic
Cubic
Relative levels
Relative levels
PMI (d)
PMI (d)
D
25℃ 1.5 day
25℃ 4 day
mt-co1
mt-co1
28S rRNA
circFat3
circFat3
28S rRNA
(276 bp)
(276 bp)
(195 bp)
(105 bp)
(195 bp)
(105 bp)
Figure S2. Construction and validation of 25℃ PMI models for nine-week-old mice using semi-quantitative RT-PCR. (A) The postmortem levels of mt-co1, 28S rRNA, and circFat3 in brain tissue at days 0, 1, 2, 4, and 8 at 25℃. (B) The linear, quadratic, and cubic equations constructed based on circFat3 and mt-co1 at 25℃. (C) The linear, quadratic, and cubic equations constructed based on circFat3 and 28S rRNA at 25℃. (D) The electrophoresis results of validation samples at 1.5 and 4 days for 25℃ models. The grouping of gels cropped from different gel was divided by the white space. The standard sample was used to eliminate the influence of exposure conditions.
