# Supplementary material for: Circular RNA circFat3 as a biomarker for construction of postmortem interval Estimation models in mouse brain tissues at multiple temperatures
Source: Sci Rep. 2025 Jul 1;15:21577. doi: 10.1038/s41598-025-07998-0 (PMC12216475; doi:10.1038/s41598-025-07998-0)
Supplement: Supplementary file 3 — Supplementary Material 3 [file 41598_2025_7998_MOESM3_ESM.pdf]

Figure 1

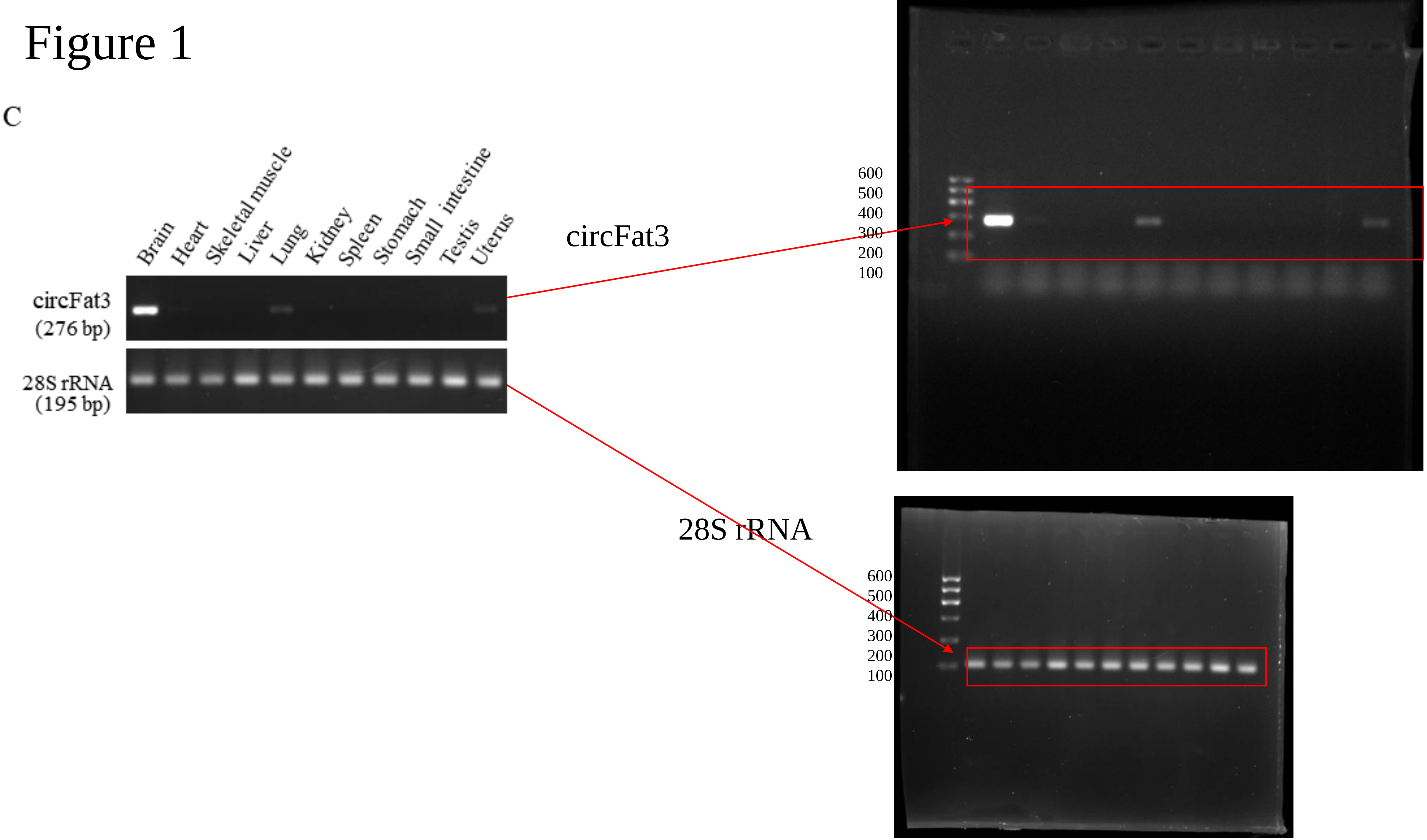

C
Brain
Heart
Skeletal muscle
Liver
Lung
Kidney
Spleen
Stomach
Small intestine
Testis
Uterus
circFat3
(276 bp)
28S rRNA
(195 bp)
circFat3
600
500
400
300
200
100
28S rRNA
600
500
400
300
200
100

Figure 2

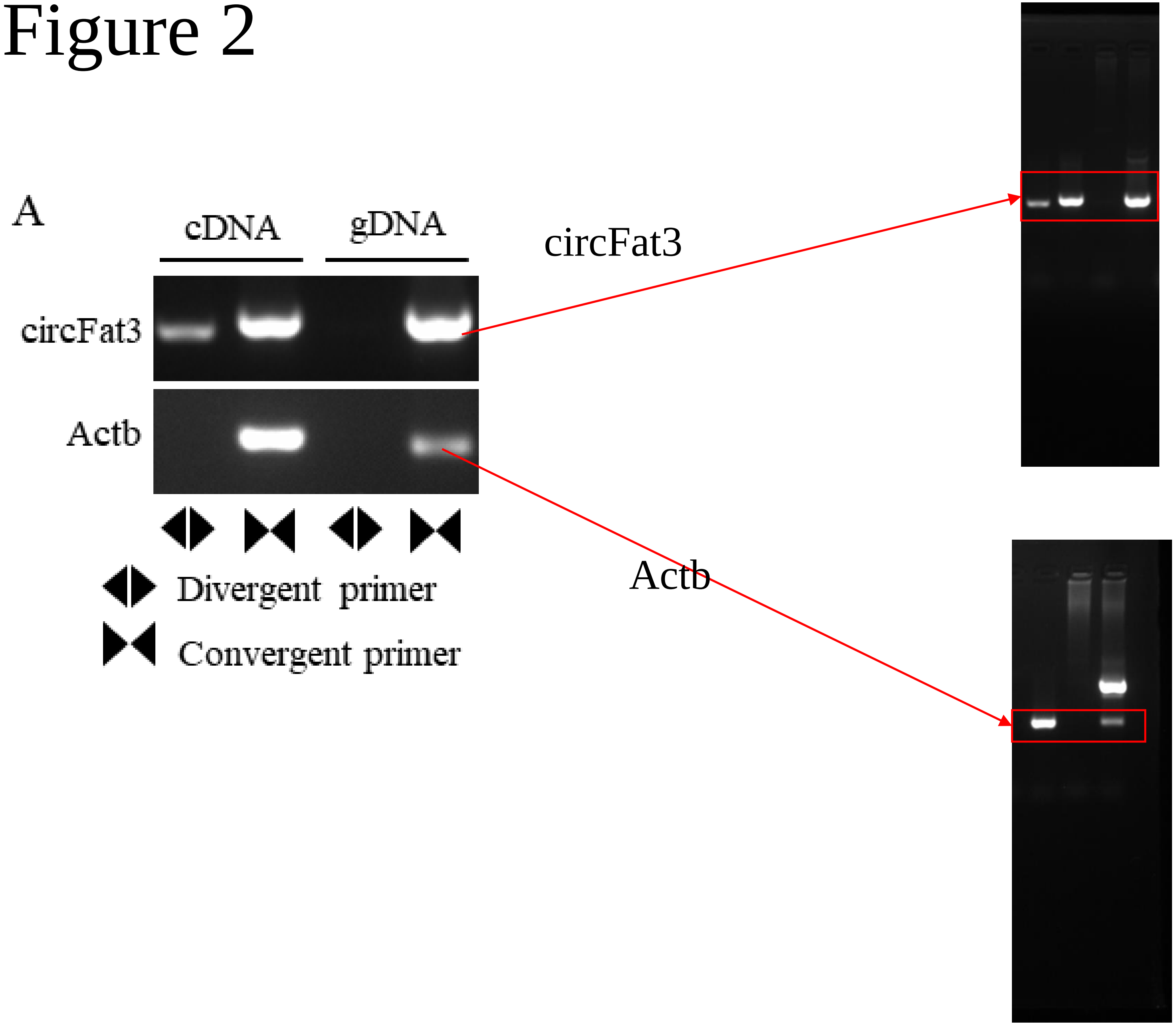
A
cDNA
gDNA
circFat3
Actb
Divergent primer
Convergent primer
circFat3
Actb

Figure 2

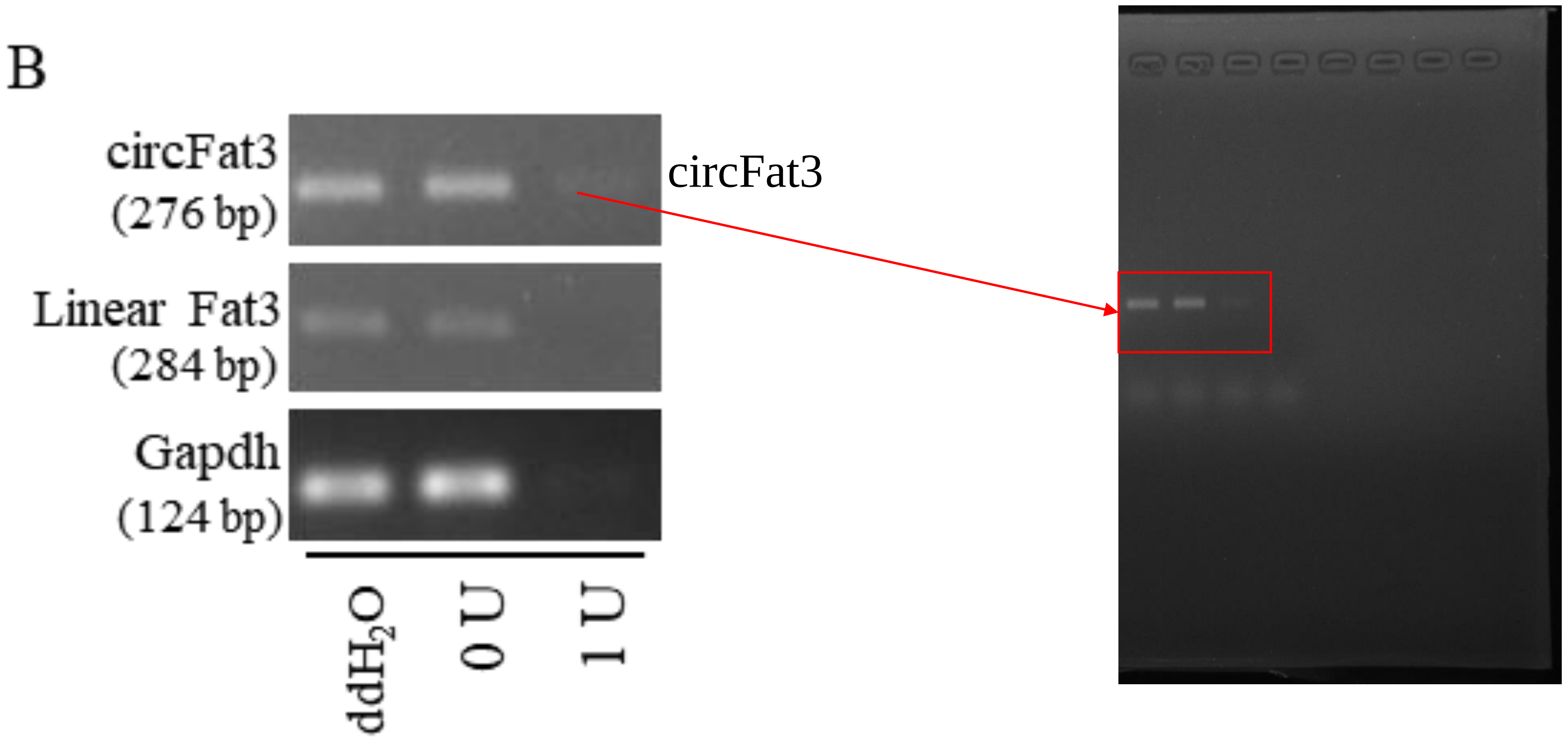
B
circFat3
(276 bp)
Linear Fat3
(284 bp)
Gapdh
(124 bp)
ddH2O
0 U
1 U
circFat3

Figure 2

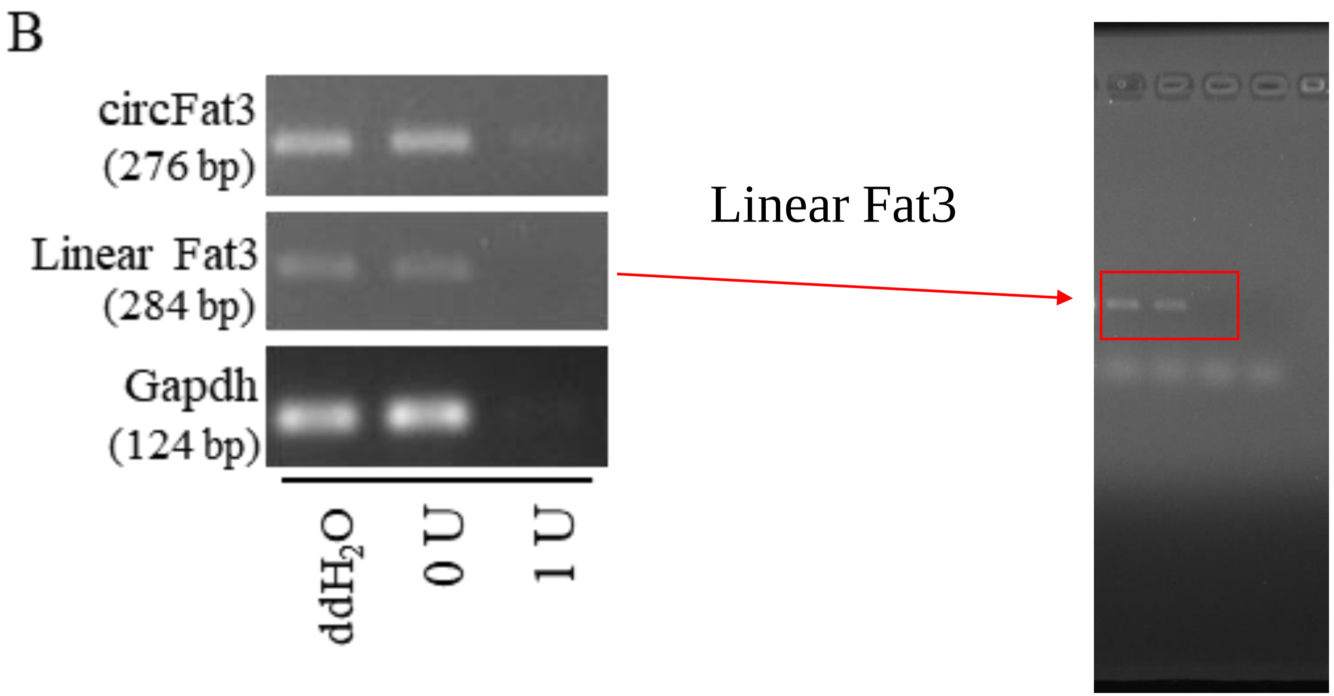
B
circFat3
(276 bp)
Linear Fat3
(284 bp)
Gapdh
(124 bp)
ddH2O
0 U
1 U
Linear Fat3

Figure 2

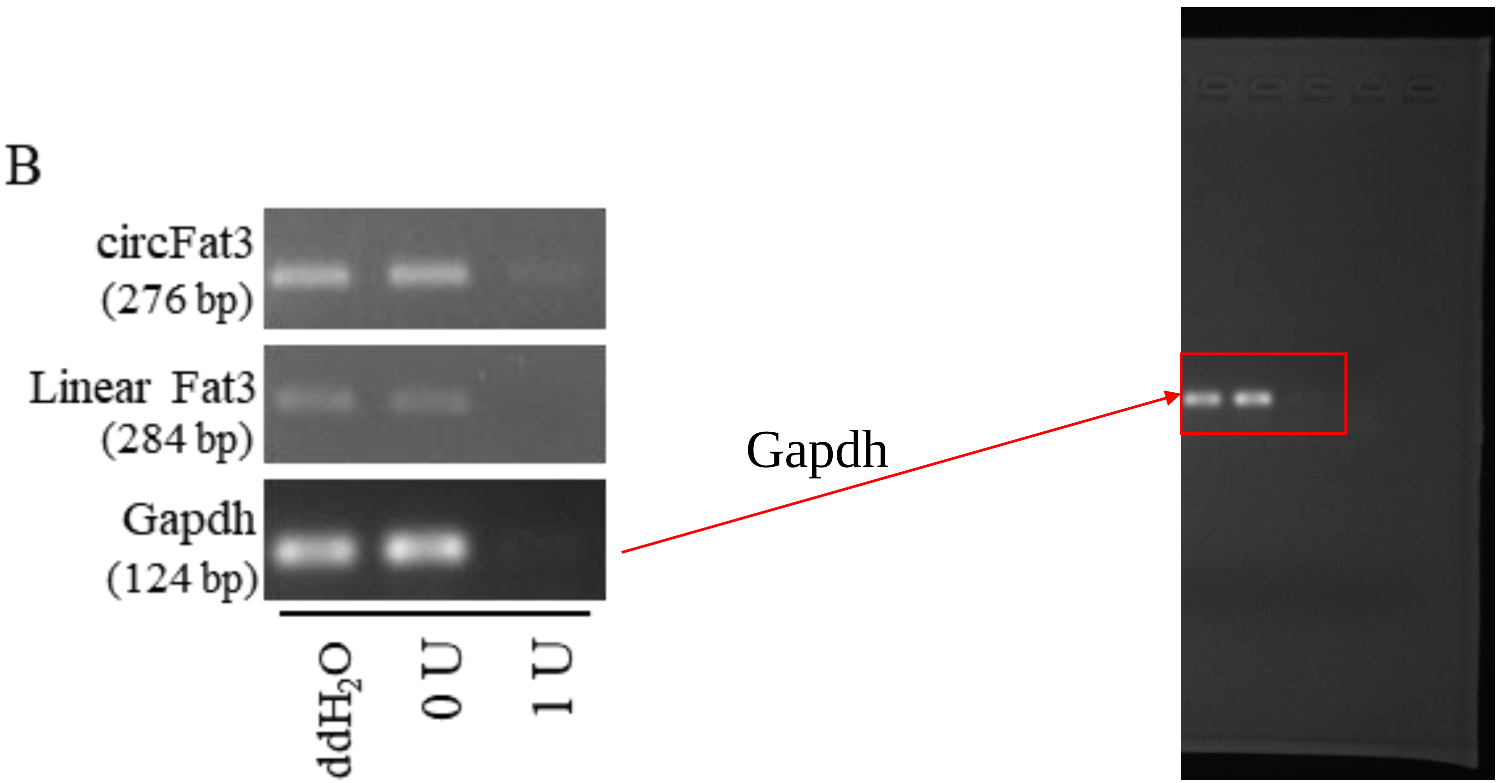
B
circFat3
(276 bp)
Linear Fat3
(284 bp)
Gapdh
(124 bp)
ddH2O
0 U
1 U
Gapdh

Figure 3

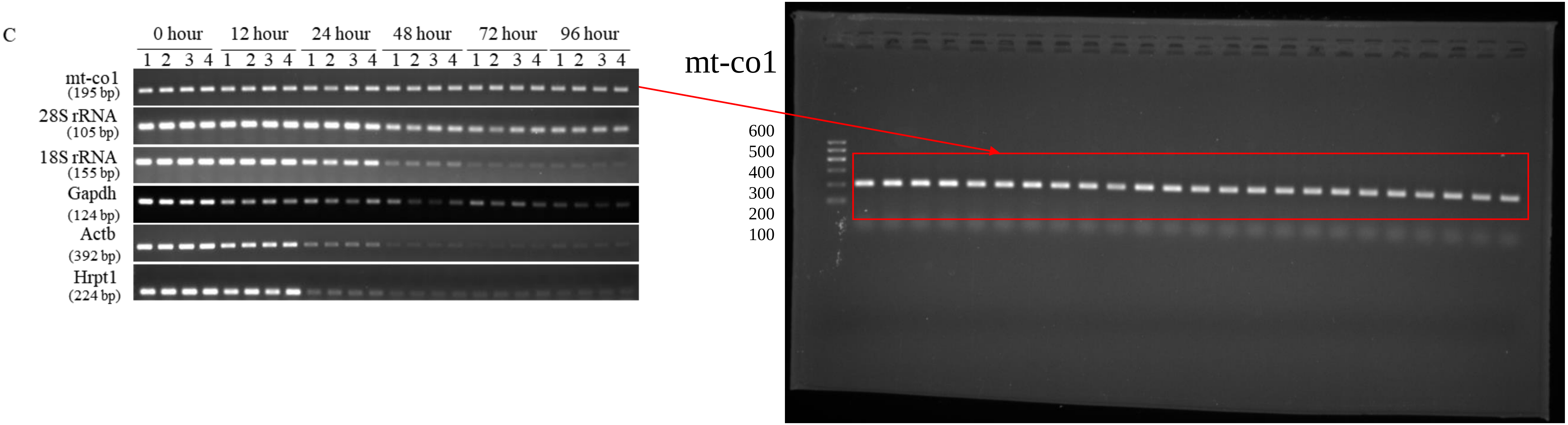

C
0 hour
12 hour
24 hour
48 hour
72 hour
96 hour
1 2 3 4 1 2 3 4 1 2 3 4 1 2 3 4 1 2 3 4 1 2 3 4
mt-co1 (195 bp)
28S rRNA (105 bp)
18S rRNA (155 bp)
Gapdh (124 bp)
Actb (392 bp)
Hrpt1 (224 bp)
mt-co1
600
500
400
300
200
100

Figure 3

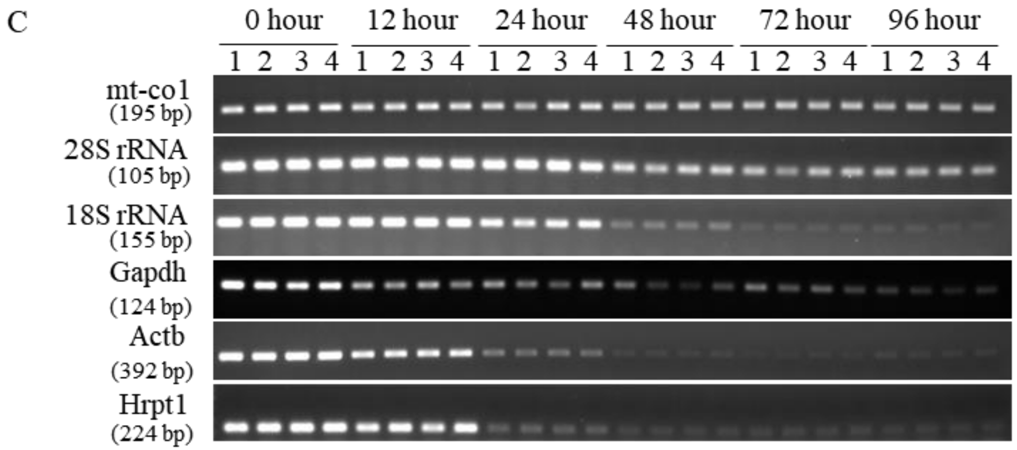

C
0 hour
12 hour
24 hour
48 hour
72 hour
96 hour
1 2 3 4 1 2 3 4 1 2 3 4 1 2 3 4 1 2 3 4 1 2 3 4
mt-co1
(195 bp)
28S rRNA
(105 bp)
18S rRNA
(155 bp)
Gapdh
(124 bp)
Actb
(392 bp)
Hrpt1
(224 bp)

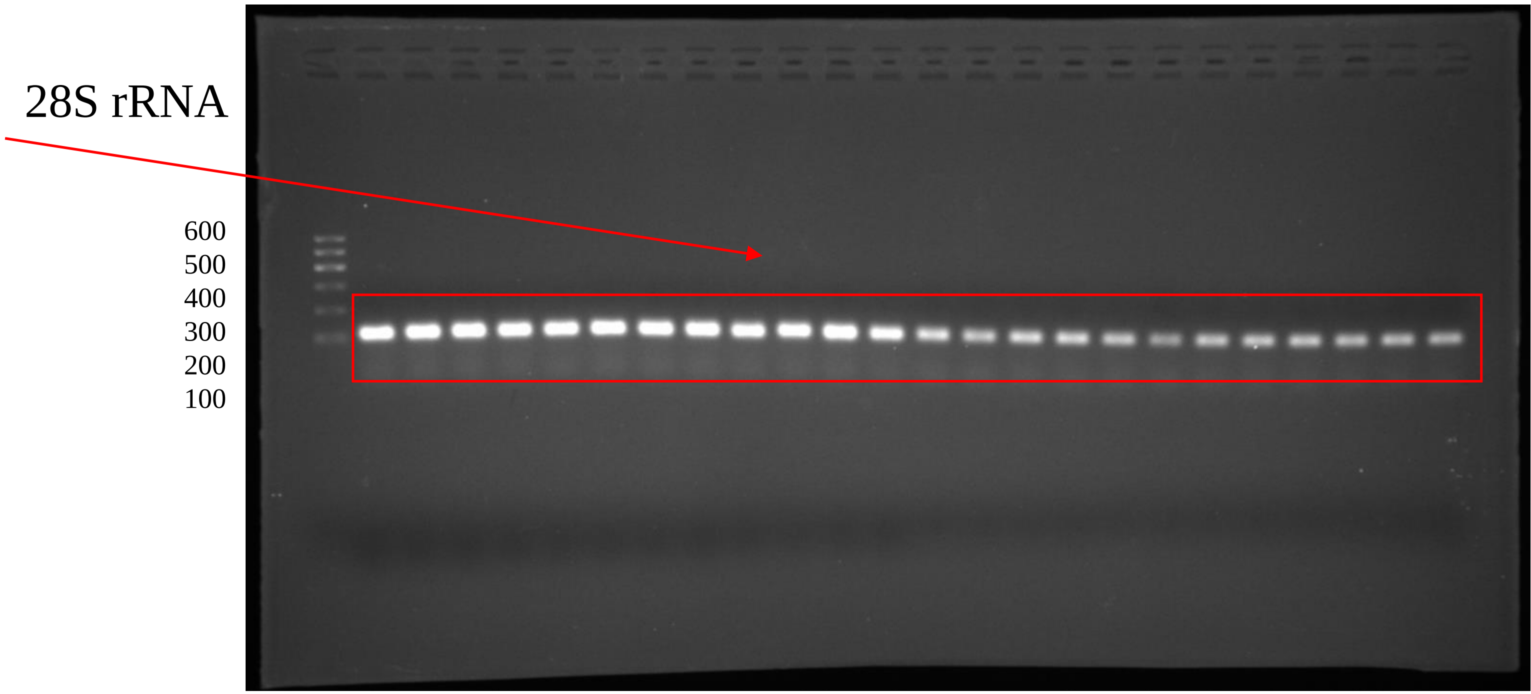

28S rRNA
600
500
400
300
200
100

Figure 3

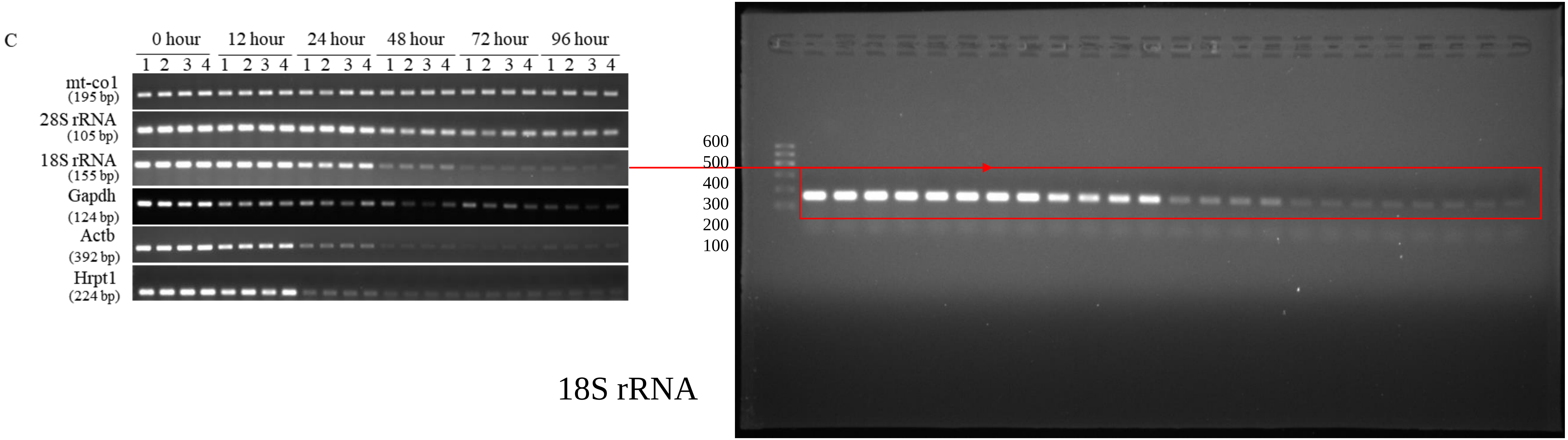

18S rRNA

Figure 3

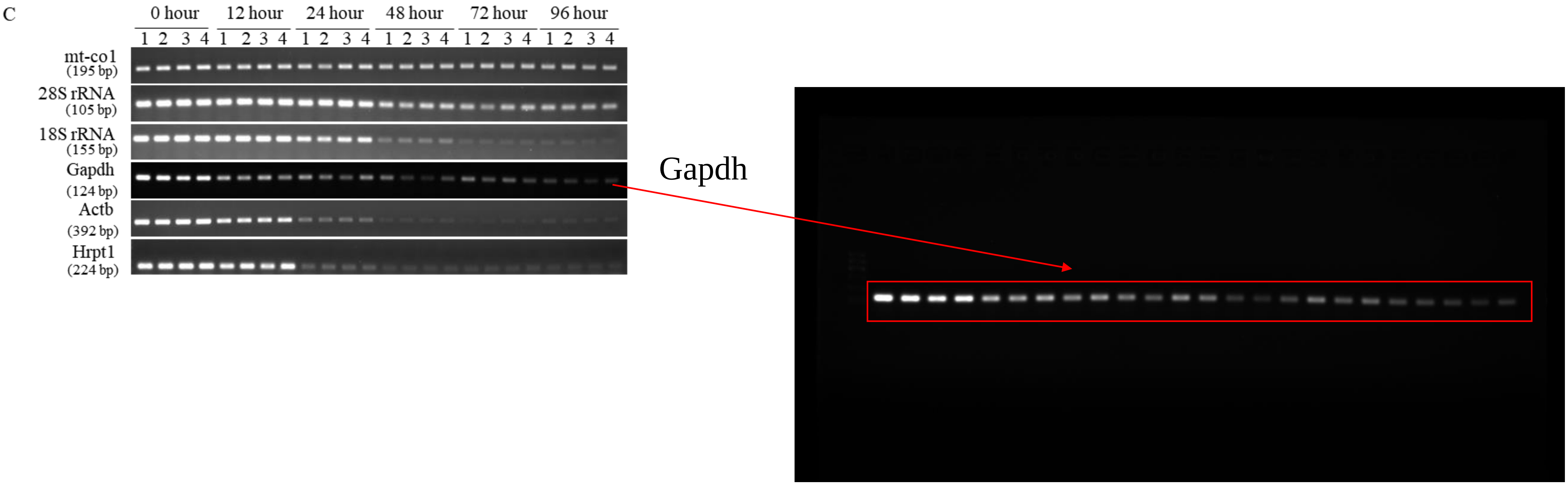
C
0 hour
12 hour
24 hour
48 hour
72 hour
96 hour
1 2 3 4 1 2 3 4 1 2 3 4 1 2 3 4 1 2 3 4 1 2 3 4
mt-co1
(195 bp)
28S rRNA
(105 bp)
18S rRNA
(155 bp)
Gapdh
(124 bp)
Actb
(392 bp)
Hrpt1
(224 bp)
Gapdh

Figure 3

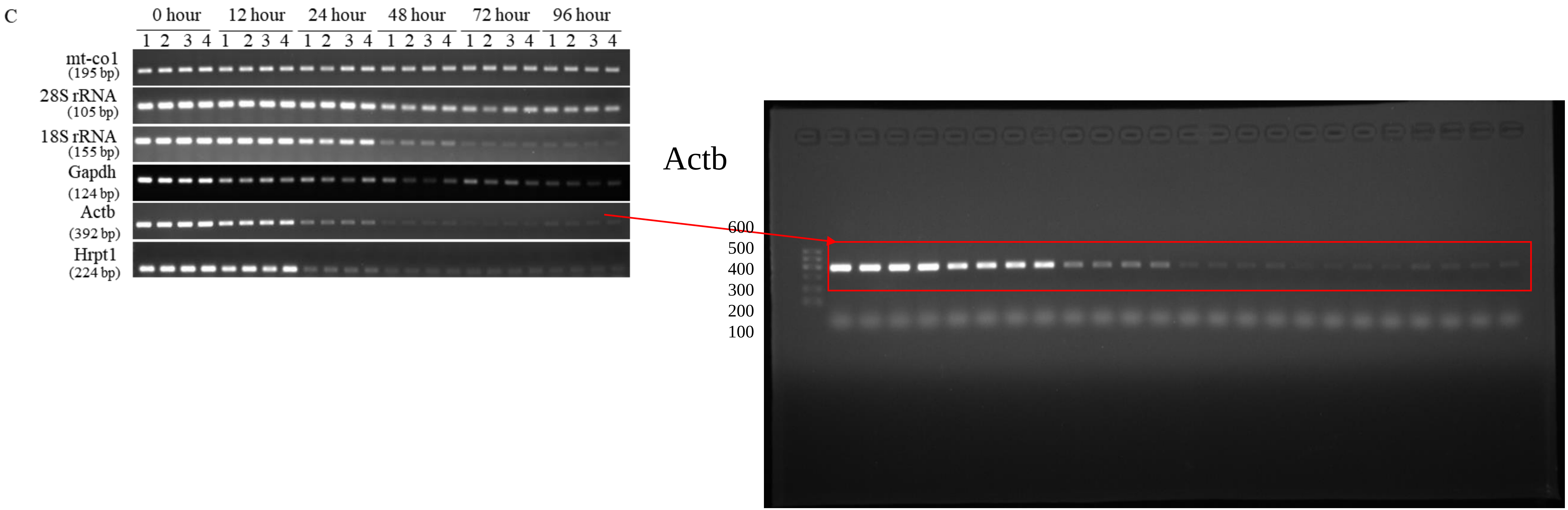
C
0 hour
12 hour
24 hour
48 hour
72 hour
96 hour
1 2 3 4 1 2 3 4 1 2 3 4 1 2 3 4 1 2 3 4 1 2 3 4
mt-co1 (195 bp)
28S rRNA (105 bp)
18S rRNA (155 bp)
Gapdh (124 bp)
Actb (392 bp)
Hrpt1 (224 bp)
Actb
600
500
400
300
200
100

Figure 3

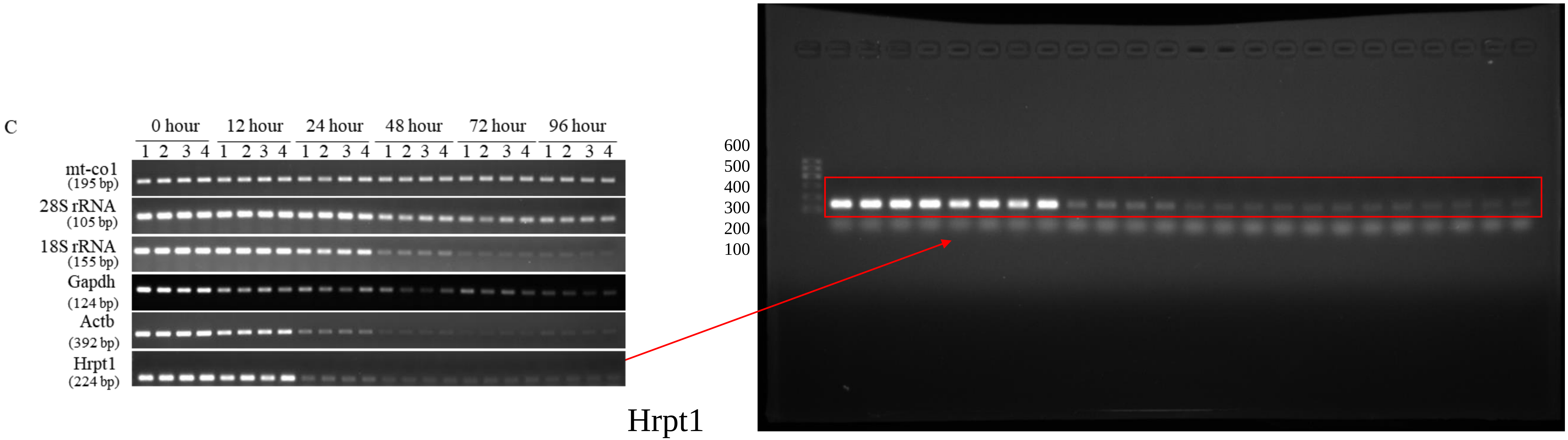

C
0 hour
12 hour
24 hour
48 hour
72 hour
96 hour
1 2 3 4 1 2 3 4 1 2 3 4 1 2 3 4 1 2 3 4 1 2 3 4
mt-co1 (195 bp)
28S rRNA (105 bp)
18S rRNA (155 bp)
Gapdh (124 bp)
Actb (392 bp)
Hrpt1 (224 bp)
Hrpt1
600
500
400
300
200
100

Figure 4

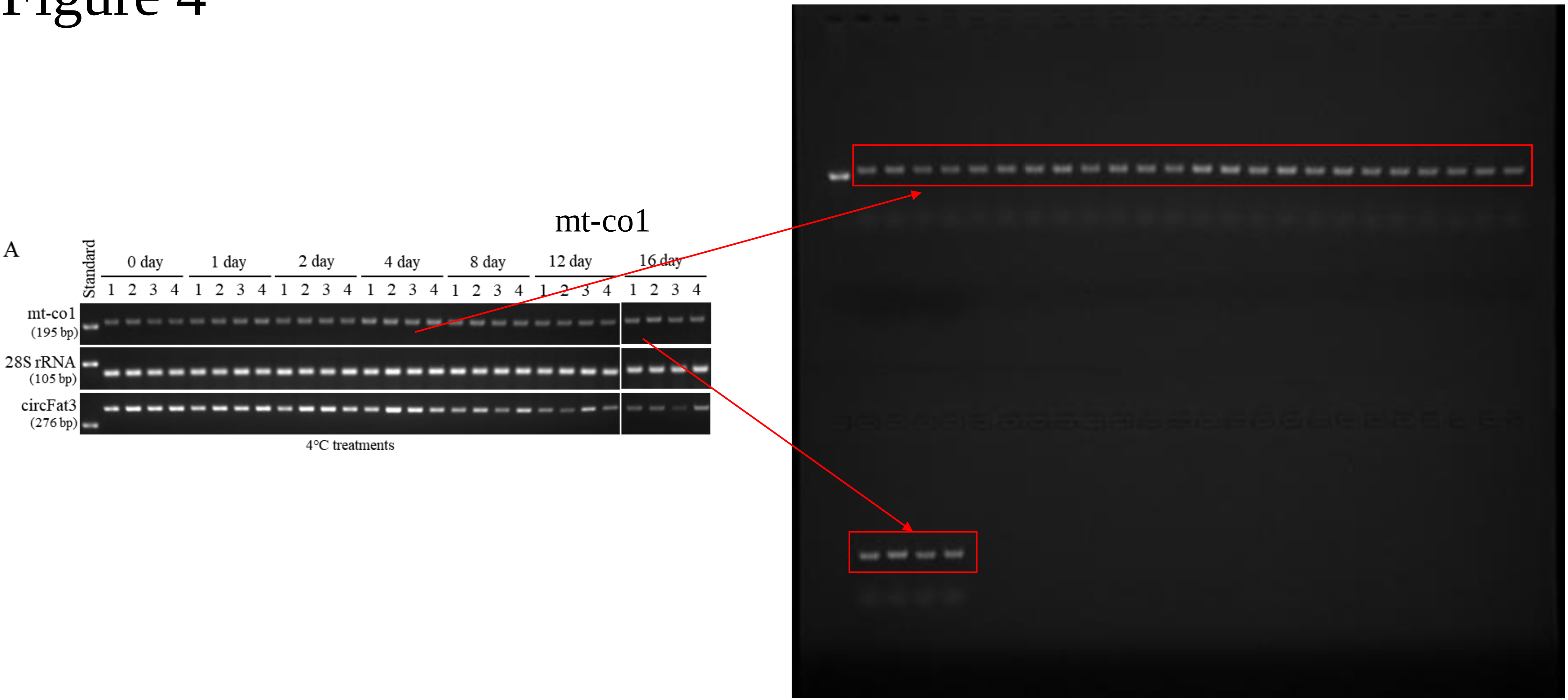
mt-co1
A
Standard
0 day
1 day
2 day
4 day
8 day
12 day
16 day
1 2 3 4 1 2 3 4 1 2 3 4 1 2 3 4 1 2 3 4 1 2 3 4 1 2 3 4
mt-co1
(195 bp)
28S rRNA
(105 bp)
circFat3
(276 bp)
4°C treatments

Figure 4

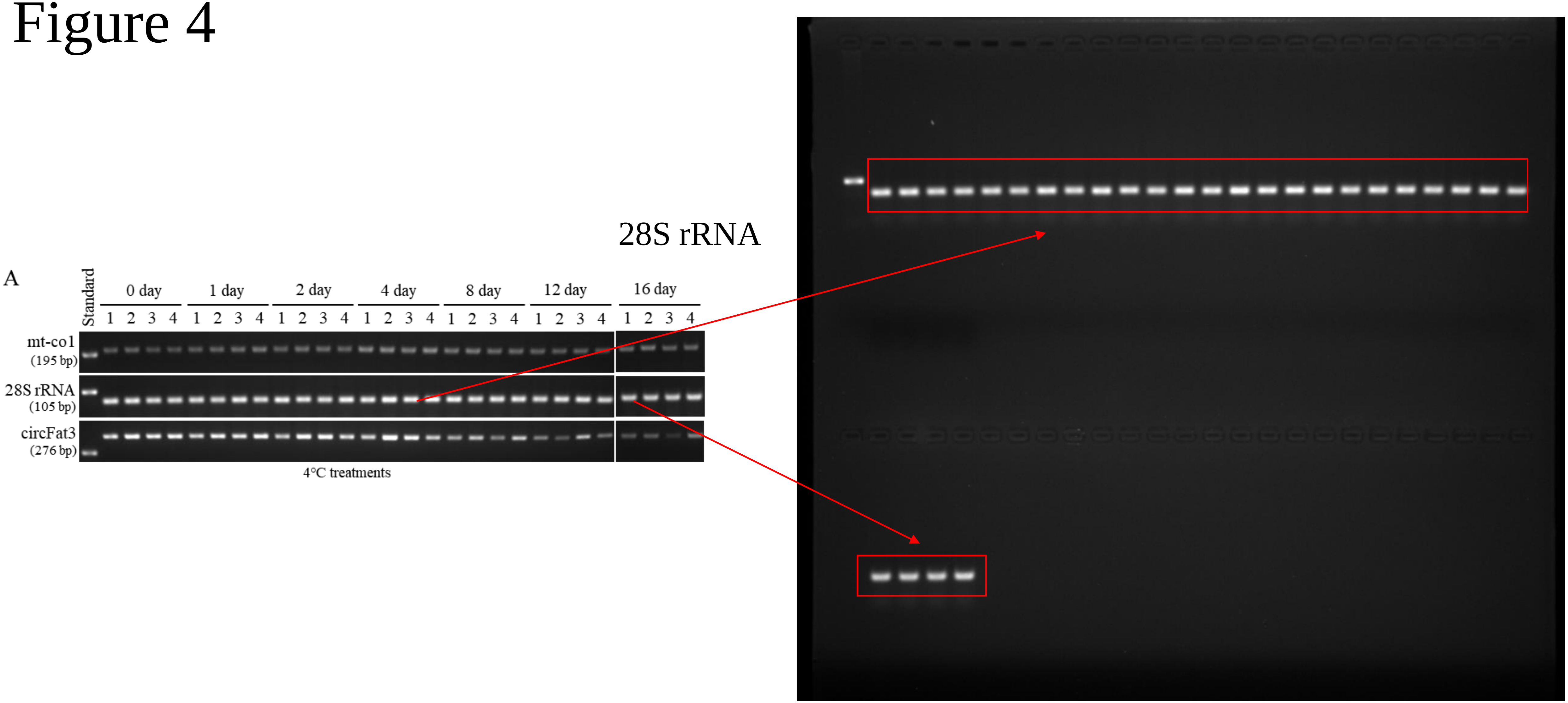

28S rRNA
A
Standard
0 day
1 day
2 day
4 day
8 day
12 day
16 day
1 2 3 4 1 2 3 4 1 2 3 4 1 2 3 4 1 2 3 4 1 2 3 4 1 2 3 4
mt-co1
(195 bp)
28S rRNA
(105 bp)
circFat3
(276 bp)
4°C treatments

Figure 4

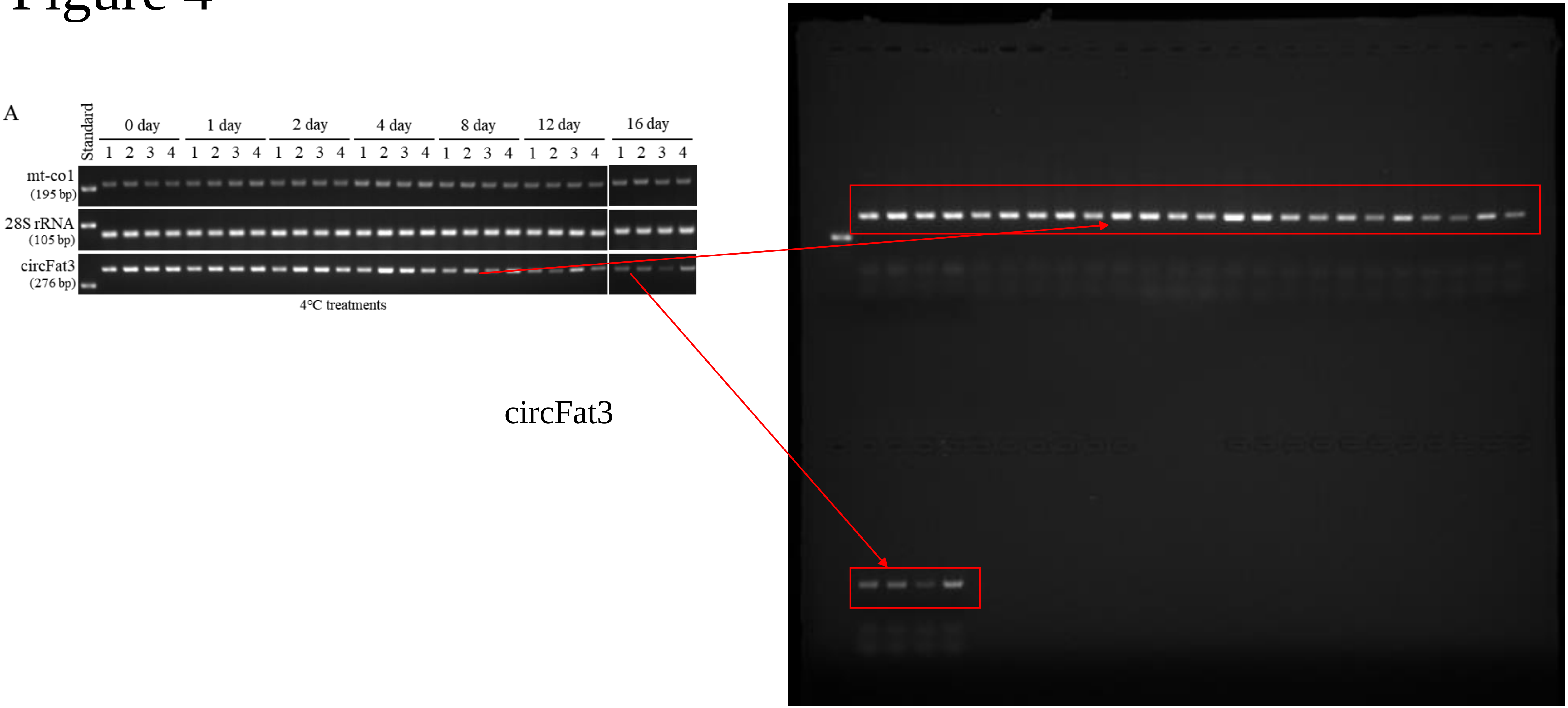

A
Standard
0 day
1 day
2 day
4 day
8 day
12 day
16 day
1 2 3 4 1 2 3 4 1 2 3 4 1 2 3 4 1 2 3 4 1 2 3 4 1 2 3 4
mt-co1 (195 bp)
28S rRNA (105 bp)
circFat3 (276 bp)
4°C treatments
circFat3

Figure 4

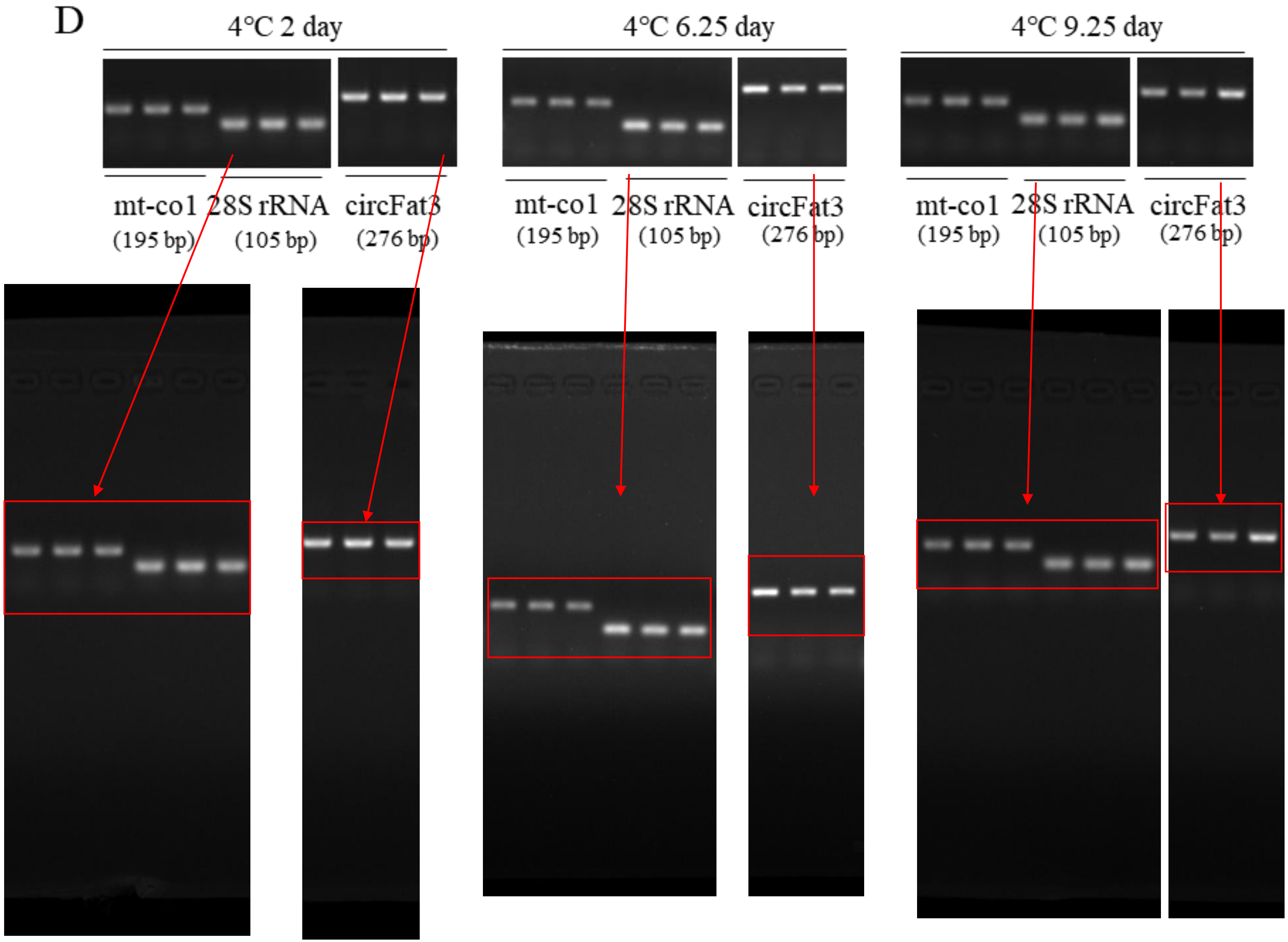
D
4°C 2 day
mt-co1
(195 bp)
28S rRNA
(105 bp)
circFat3
(276 bp)
4°C 6.25 day
mt-co1
(195 bp)
28S rRNA
(105 bp)
circFat3
(276 bp)
4°C 9.25 day
mt-co1
(195 bp)
28S rRNA
(105 bp)
circFat3
(276 bp)

Figure 5

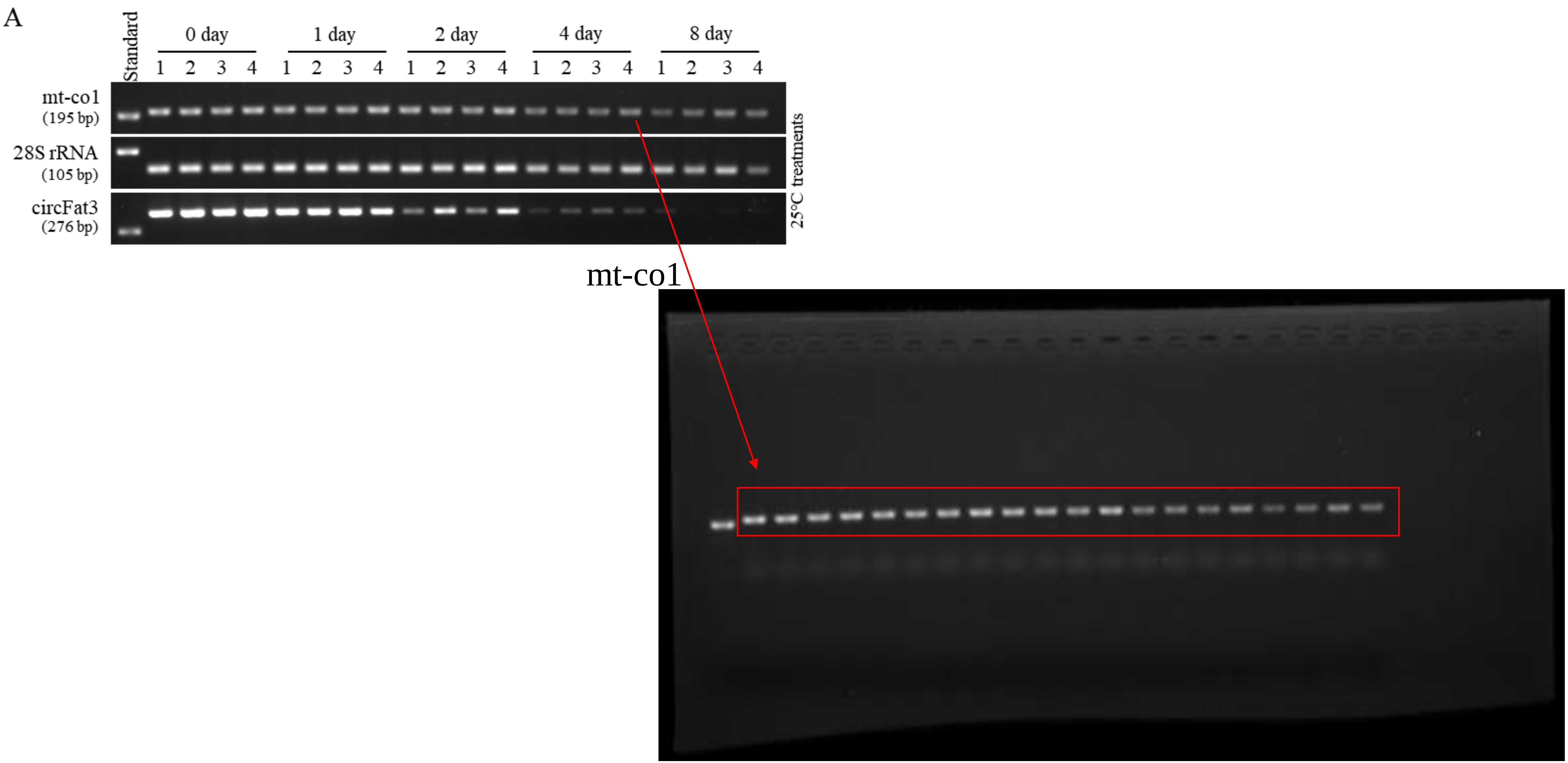
A
Standard
0 day
1 day
2 day
4 day
8 day
1 2 3 4 1 2 3 4 1 2 3 4 1 2 3 4 1 2 3 4
mt-co1
(195 bp)
28S rRNA
(105 bp)
circFat3
(276 bp)
25°C treatments
mt-co1

Figure 5

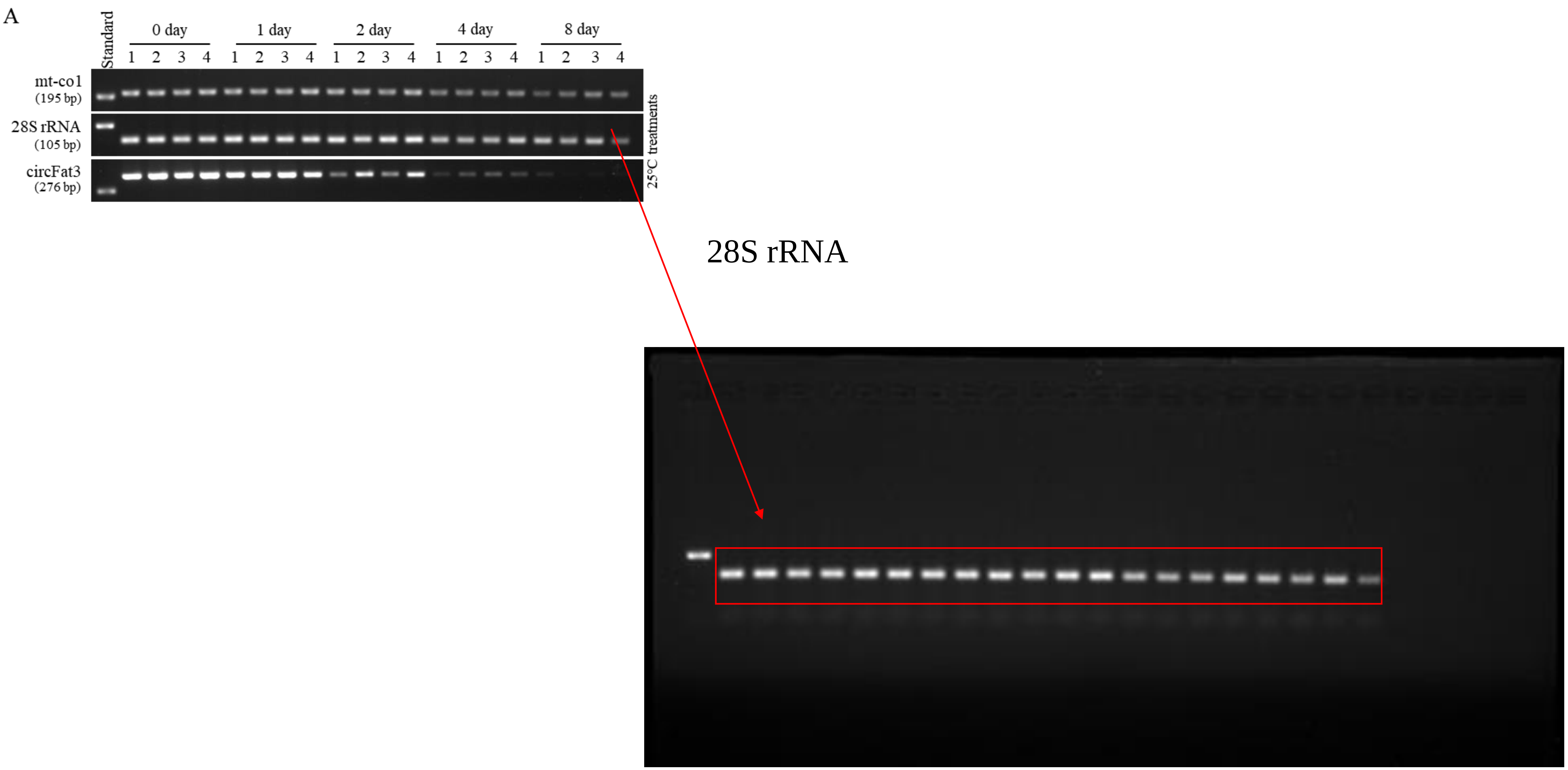
A
Standard
0 day
1 day
2 day
4 day
8 day
1 2 3 4 1 2 3 4 1 2 3 4 1 2 3 4 1 2 3 4
mt-co1
(195 bp)
28S rRNA
(105 bp)
circFat3
(276 bp)
25°C treatments
28S rRNA

Figure 5

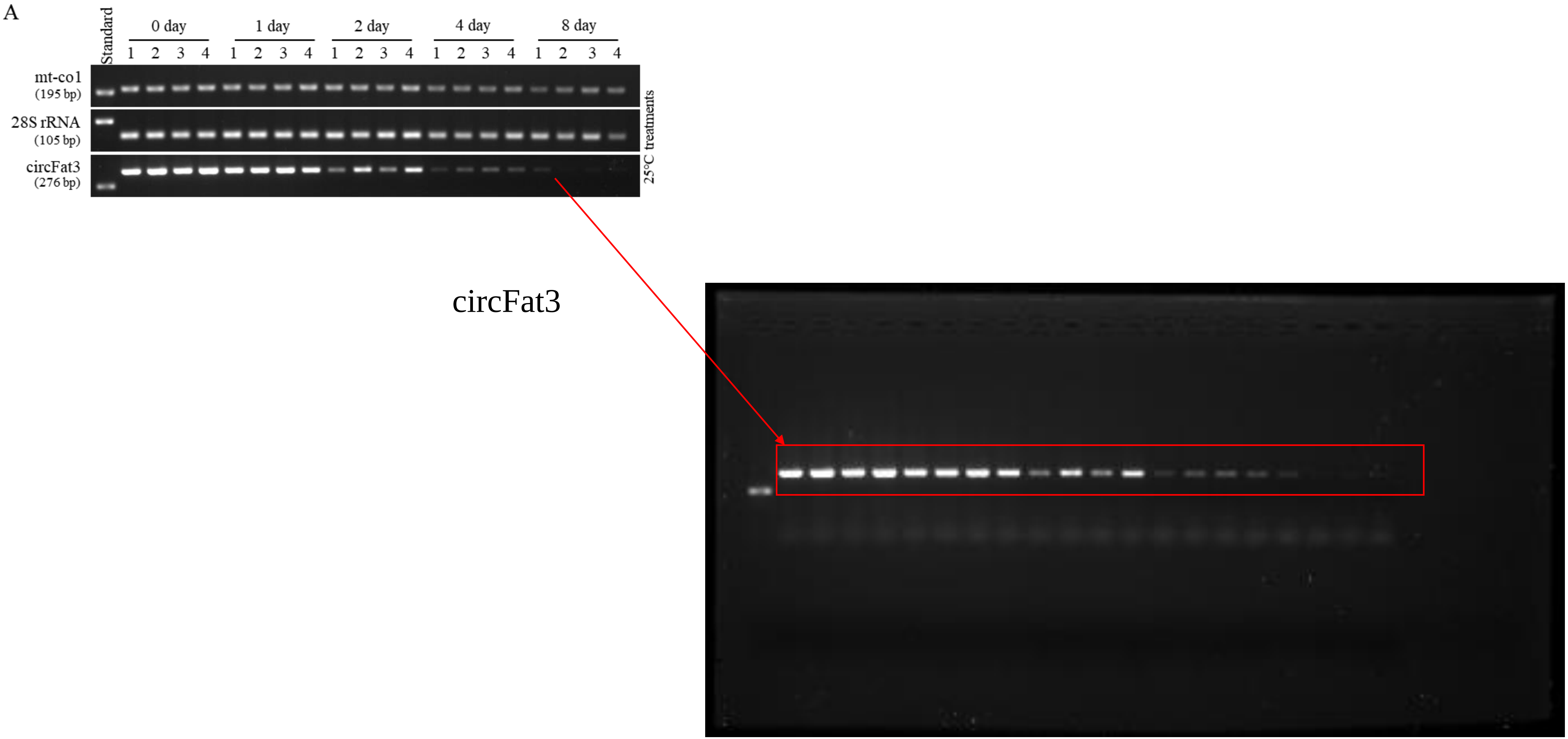
A
Standard
0 day
1 day
2 day
4 day
8 day
1 2 3 4 1 2 3 4 1 2 3 4 1 2 3 4 1 2 3 4
mt-co1
(195 bp)
28S rRNA
(105 bp)
circFat3
(276 bp)
25°C treatments
circFat3

Figure 5

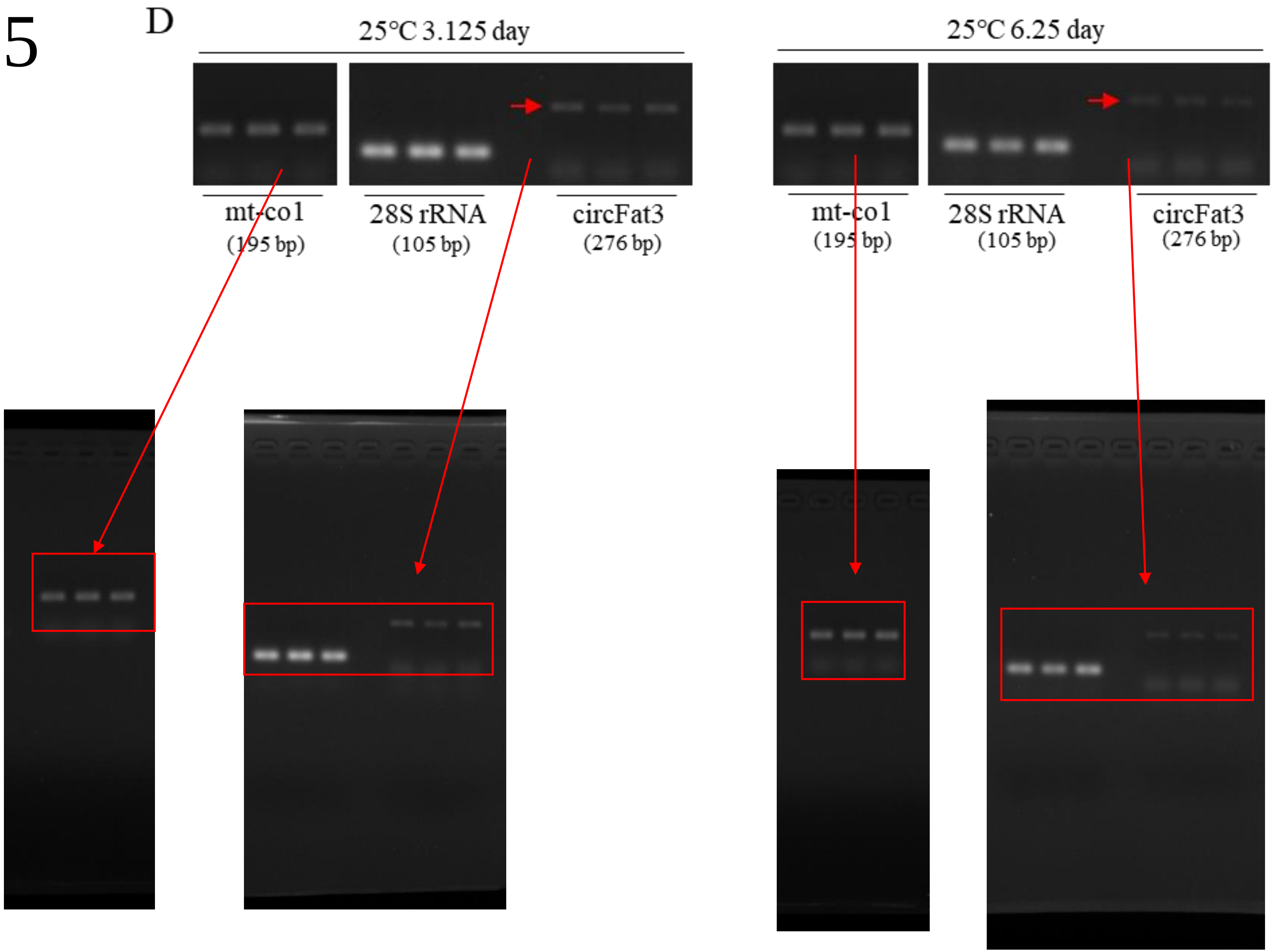

D
25°C 3.125 day
25°C 6.25 day
mt-co1
(195 bp)
28S rRNA
(105 bp)
circFat3
(276 bp)
mt-co1
(195 bp)
28S rRNA
(105 bp)
circFat3
(276 bp)

Figure 6

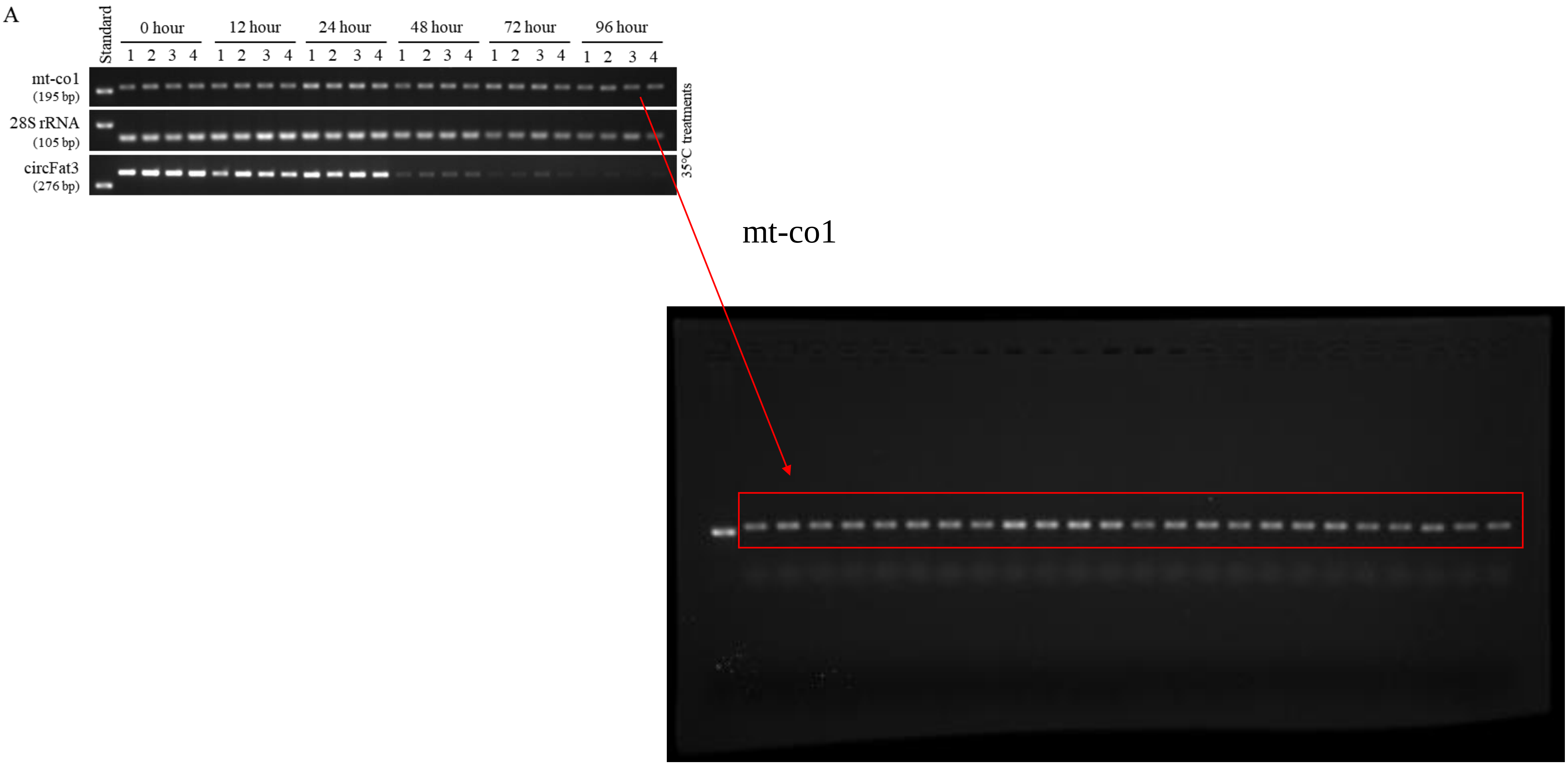
A
Standard
0 hour
12 hour
24 hour
48 hour
72 hour
96 hour
1 2 3 4 1 2 3 4 1 2 3 4 1 2 3 4 1 2 3 4 1 2 3 4
mt-co1
(195 bp)
28S rRNA
(105 bp)
circFat3
(276 bp)
35°C treatments
mt-co1

Figure 6

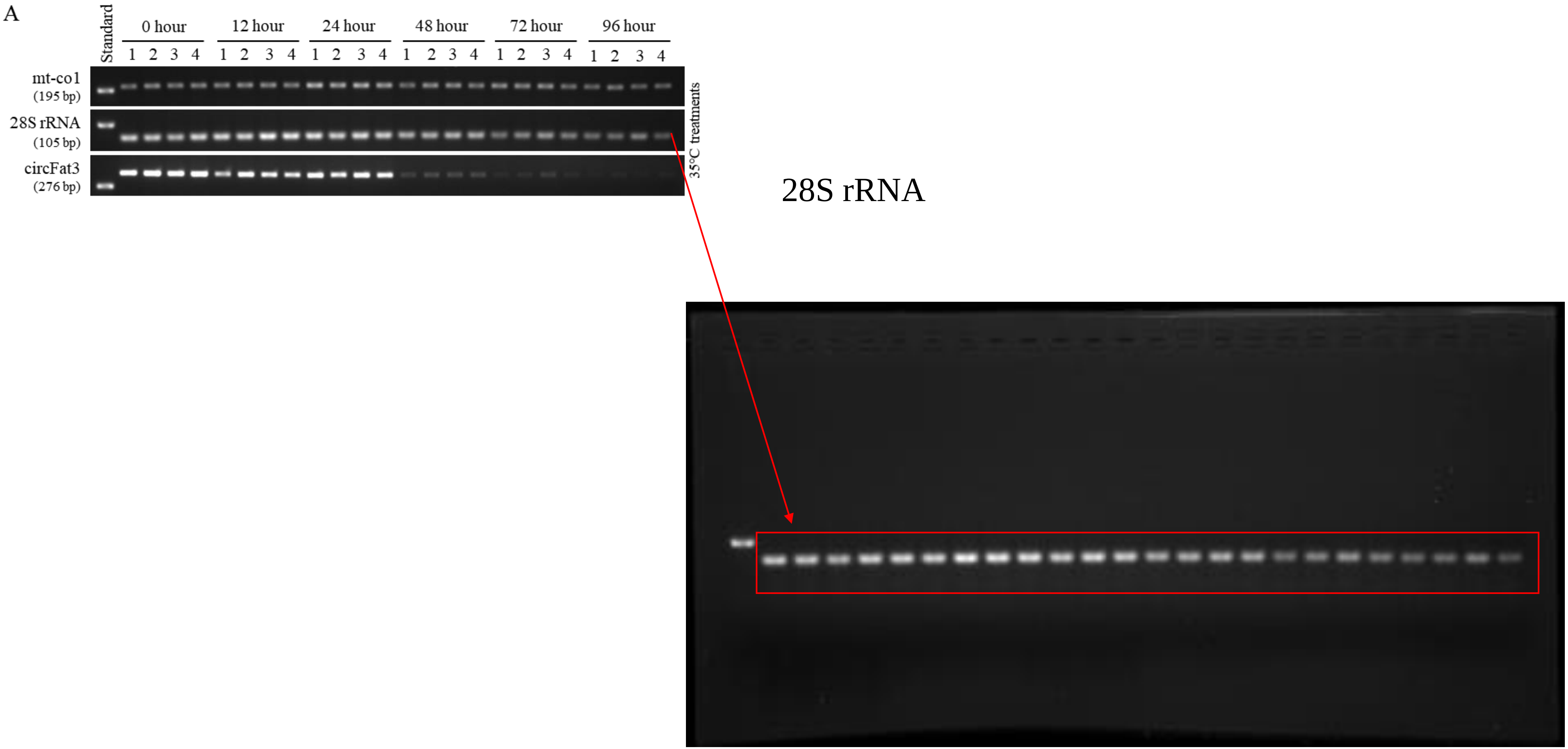
A
Standard
0 hour
12 hour
24 hour
48 hour
72 hour
96 hour
1 2 3 4 1 2 3 4 1 2 3 4 1 2 3 4 1 2 3 4 1 2 3 4
mt-co1
(195 bp)
28S rRNA
(105 bp)
circFat3
(276 bp)
35°C treatments
28S rRNA

Figure 6

A

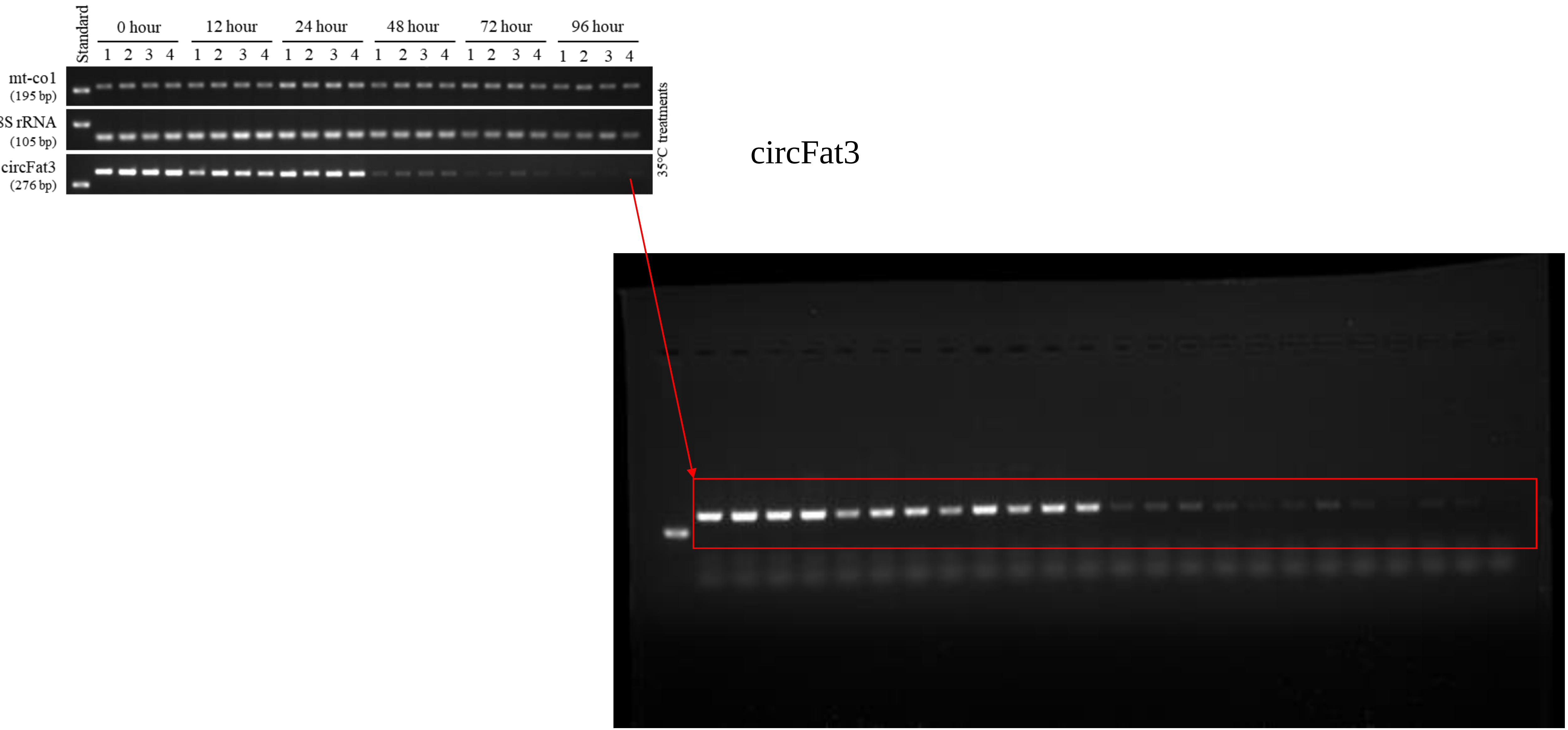
Standard
0 hour
12 hour
24 hour
48 hour
72 hour
96 hour
1 2 3 4 1 2 3 4 1 2 3 4 1 2 3 4 1 2 3 4 1 2 3 4
mt-co1
(195 bp)
28S rRNA
(105 bp)
circFat3
(276 bp)
35°C treatments
circFat3

Figure 6

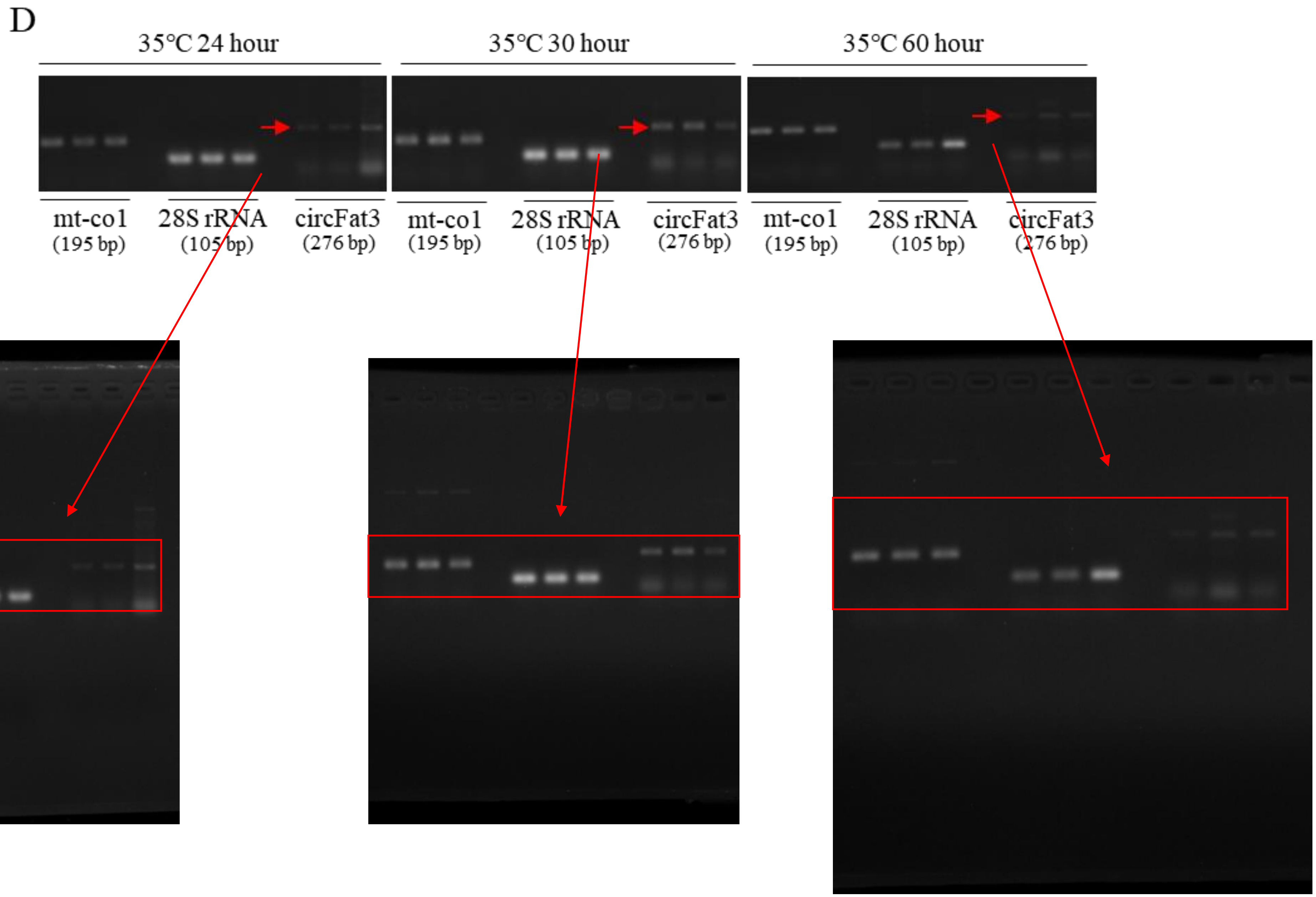
D
35°C 24 hour
35°C 30 hour
35°C 60 hour
mt-co1
(195 bp)
28S rRNA
(105 bp)
circFat3
(276 bp)
mt-co1
(195 bp)
28S rRNA
(105 bp)
circFat3
(276 bp)
mt-co1
(195 bp)
28S rRNA
(105 bp)
circFat3
(276 bp)

Figure S2

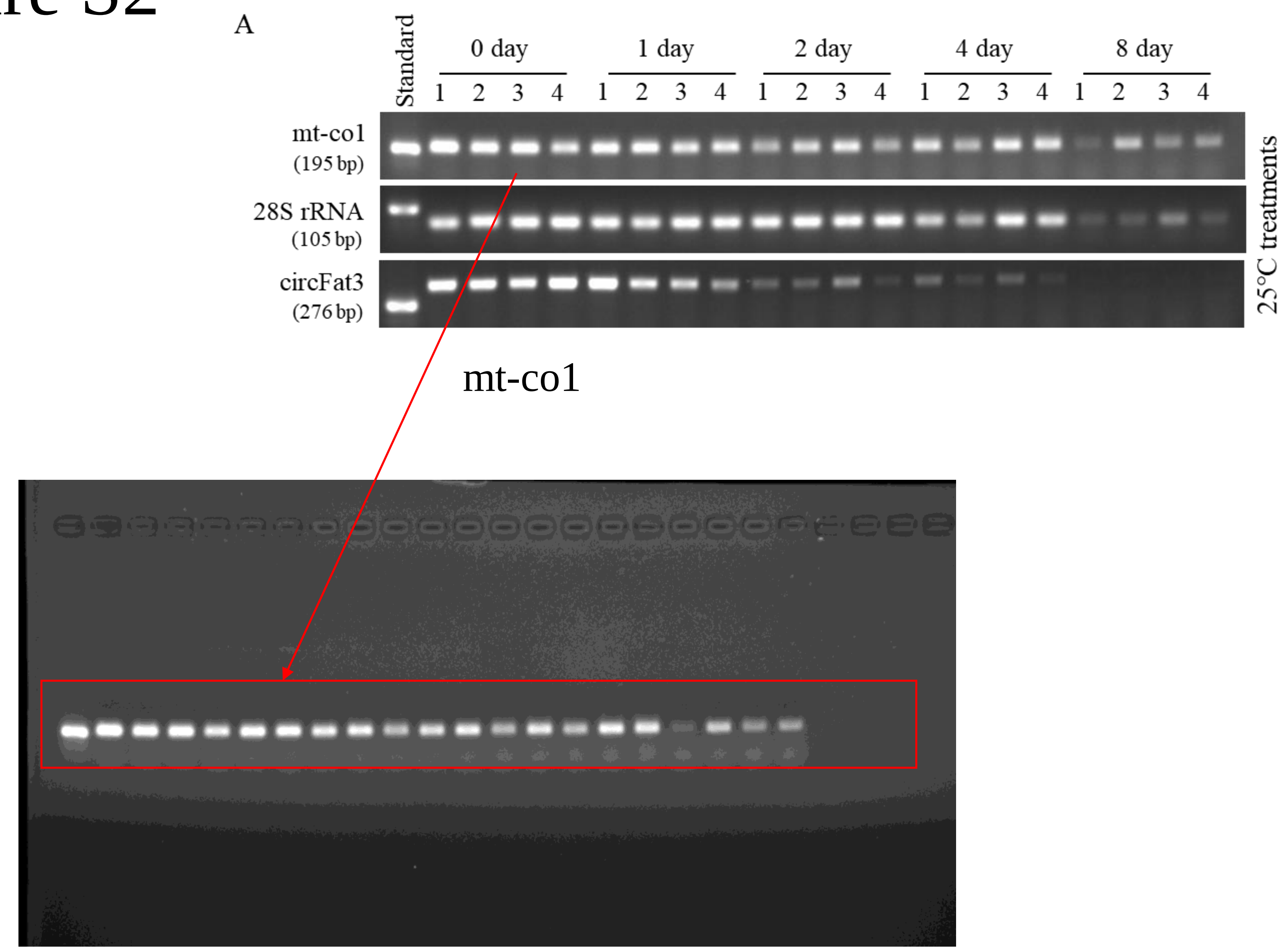

A
Standard
0 day
1 day
2 day
4 day
8 day
1 2 3 4 1 2 3 4 1 2 3 4 1 2 3 4 1 2 3 4
mt-co1
(195 bp)
28S rRNA
(105 bp)
circFat3
(276 bp)
25°C treatments
mt-co1

Figure S2

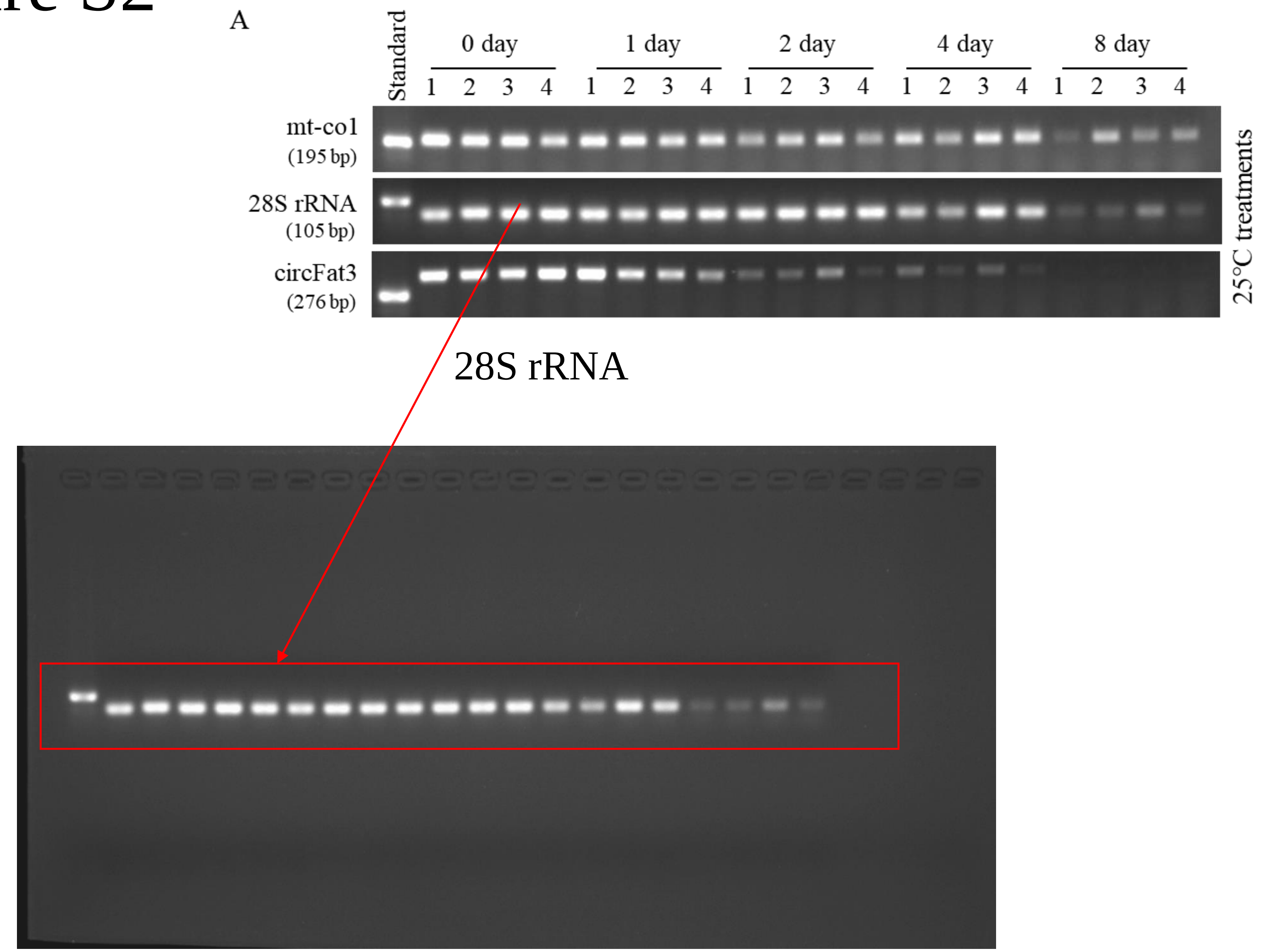
A
Standard
0 day
1 day
2 day
4 day
8 day
1 2 3 4 1 2 3 4 1 2 3 4 1 2 3 4 1 2 3 4
mt-co1
(195 bp)
28S rRNA
(105 bp)
circFat3
(276 bp)
25°C treatments
28S rRNA

Figure S2

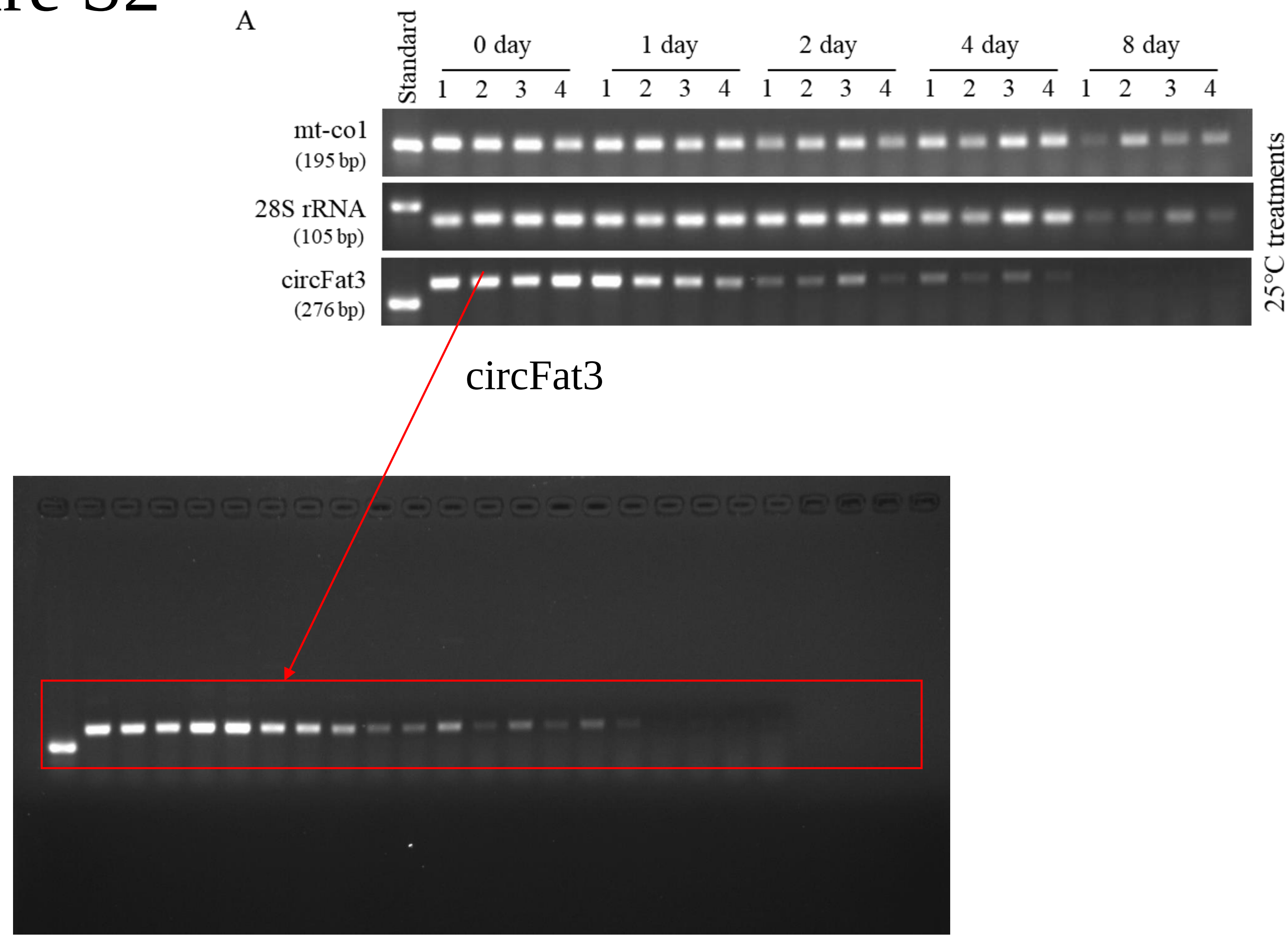
A
Standard
0 day
1 day
2 day
4 day
8 day
1 2 3 4 1 2 3 4 1 2 3 4 1 2 3 4 1 2 3 4
mt-co1
(195 bp)
28S rRNA
(105 bp)
circFat3
(276 bp)
25°C treatments
circFat3

Figure S2

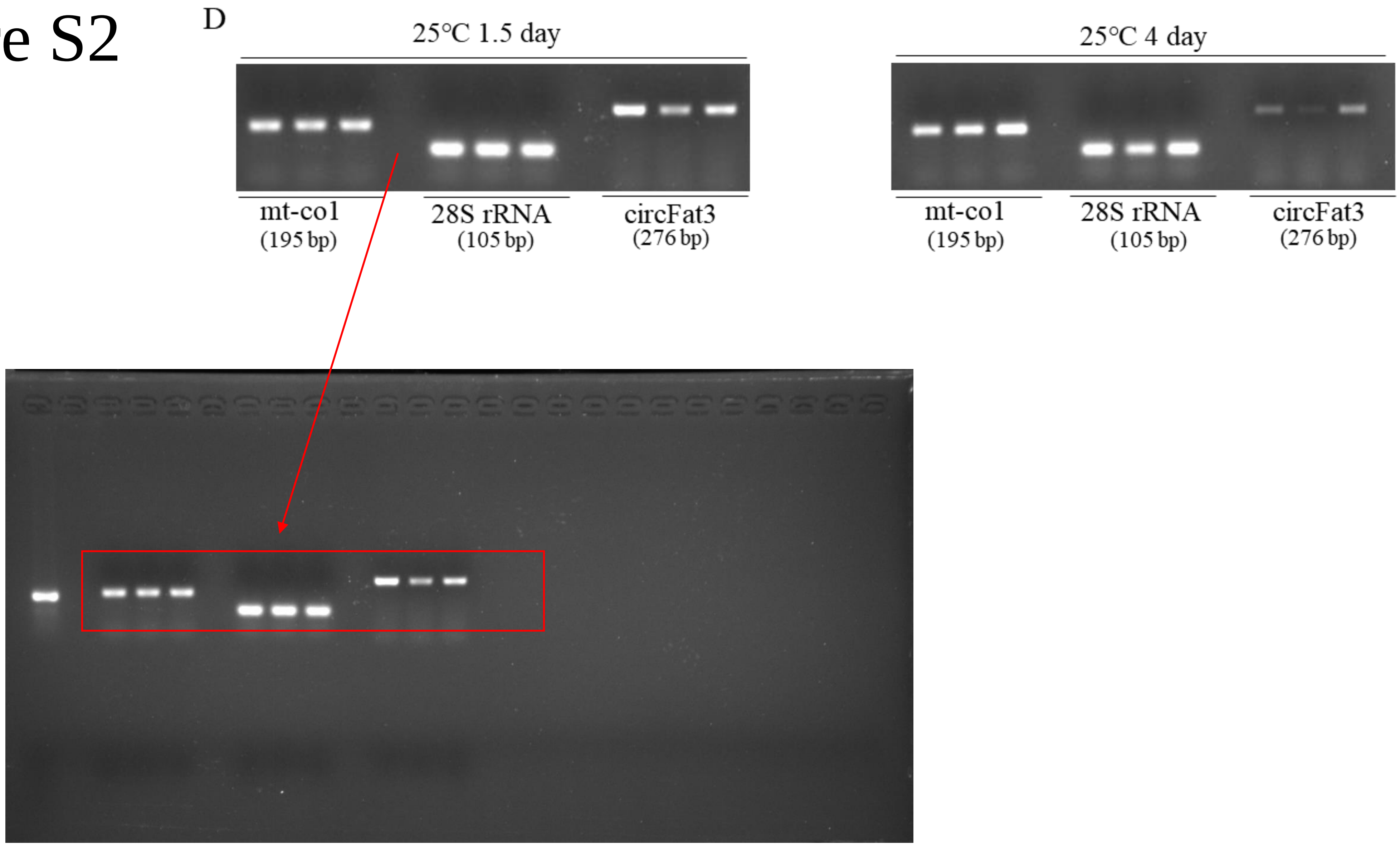
D
25℃ 1.5 day
25℃ 4 day
mt-co1
(195 bp)
28S rRNA
(105 bp)
circFat3
(276 bp)
mt-co1
(195 bp)
28S rRNA
(105 bp)
circFat3
(276 bp)

Figure S2

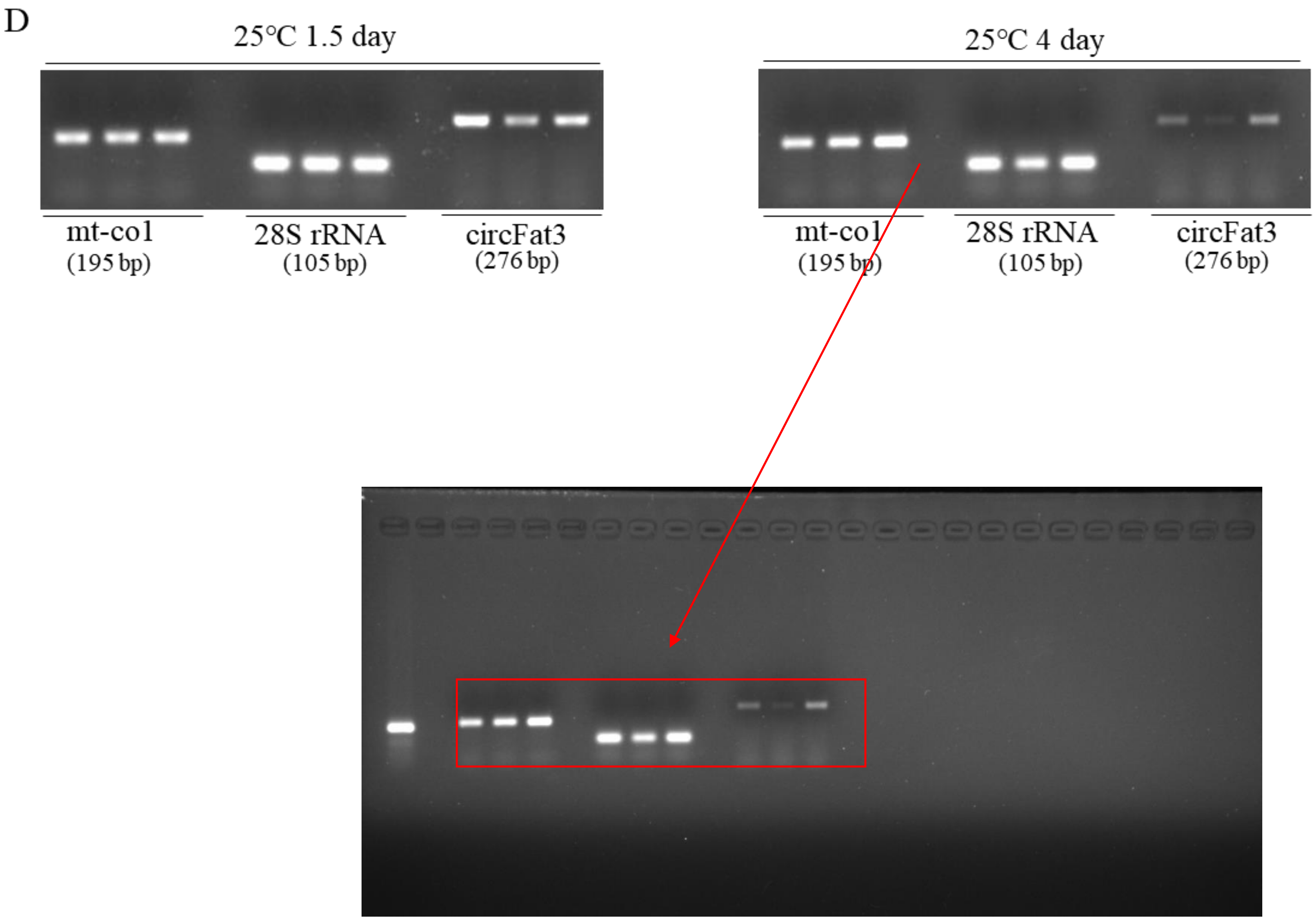
D
25°C 1.5 day
25°C 4 day
mt-co1
(195 bp)
28S rRNA
(105 bp)
circFat3
(276 bp)
mt-co1
(195 bp)
28S rRNA
(105 bp)
circFat3
(276 bp)
